# Immune Exhaustion in Chronic Infection and Cancer: Signaling Pathways and Therapeutic Interventions

**DOI:** 10.1002/mco2.70635

**Published:** 2026-02-26

**Authors:** Yali Song, Yazhi Mo, Si Chen, Yuemei Chen, Chunying Zhang, Shanying Deng, Juan Liao, Yi He, Wei Wang, Weidong Zheng, Tingting Zeng

**Affiliations:** ^1^ Department of Laboratory Medicine Shenzhen University General Hospital Shenzhen China; ^2^ Department of Laboratory Medicine, West China Hospital Sichuan University, Sichuan Clinical Research Center For Laboratory Medicine, Clinical Laboratory Medicine Research Center of West China Hospital Chengdu China; ^3^ Gastroenterology and Urology Department II, Hunan Cancer Hospital/the Affiliated Cancer Hospital of Xiangya School of Medicine Central South University; Clinical Research Center for Gastrointestinal Cancer in Hunan Province Hunan China

**Keywords:** cancer, CAR‐T cell therapy, checkpoint blockade, chronic infection, immune exhaustion

## Abstract

Immune exhaustion is a state of sustained lymphocyte dysfunction that occurs following chronic antigenic stimulation and constitutes a shared hallmark of chronic infection and cancer. Beyond being a passive consequence of persistent antigen exposure, it actively drives tumor progression by fostering immunosuppressive microenvironments. Pathogens that evade immune detection to establish chronic infection can directly induce immune exhaustion through sustained inflammatory signaling, thereby crippling cytotoxic T cell‐mediated tumor surveillance. This impairment facilitates both de novo tumorigenesis and the aggressive evolution of pre‐existing malignancies. This comprehensive review delineates the mechanisms and characteristics of immune exhaustion within the contexts of chronic infection and cancer, as well as its impact on disease progression. Furthermore, we propose a chronic infection–exhaustion–tumor axis and analyze this pathway with reference to specific pathogens. Finally, we provide a critical appraisal of current strategies designed to reverse immune exhaustion and discuss their therapeutic potential and limitations within three defined contexts: chronic infection, cancer, and the interplay between chronic infection and tumor development. By integrating insights from virology and immuno‐oncology, this work proposes therapeutic strategies to disrupt the infection–exhaustion–tumor axis, offering a roadmap for precision oncology.

## Graphical abstract

Immune exhaustion is a well‐established hallmark of both cancer and chronic infection. This review proposes a novel “infection–exhaustion–tumor axis” framework, wherein chronic pathogens (e.g., oncogenic viruses) evade immune surveillance by hijacking inflammatory signaling to drive immune cell exhaustion, thereby creating immunosuppressive niches conducive to tumor initiation and progression. This work redefines chronic infection as a modifiable risk factor for precision oncology, offering an actionable target, reversing exhaustion, to disrupt the vicious cycle between pathogen persistence and tumor evolution, contributing to tumor therapy.

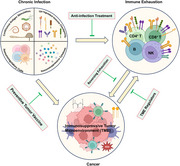

## 1 Introduction

In both chronic infection and cancer, persistent antigen exposure leads to a progressive and hierarchical deterioration of T cell function, culminating in a state of exhaustion that is strongly associated with impaired pathogen clearance and tumor progression [1]. Exhausted T cells are characterized by a loss of cytotoxic capacity, reduced proliferative potential, diminished secretion of effector cytokines, and sustained overexpression of inhibitory receptors [2]. The establishment and maintenance of the exhausted phenotype are orchestrated through an intricate interplay of diverse cellular actors and molecular pathways. Although it manifests at the single‐cell level, its full implications must be understood within the broader immune microenvironment [3, 4]. Moreover, while there are similarities, CD8^+^ T cell exhaustion in chronic infection differs in key aspects from that in cancer, reflecting differences in systemic and local antigen exposure as well as distinct influences from the tumor microenvironment (TME) [5].

Growing evidence indicates that immune exhaustion, a condition traditionally linked to T cell dysfunction, extends beyond T lymphocytes to encompass B cells and natural killer (NK) cells. These exhausted populations display compromised effector functions, defective antigen presentation, reduced cytokine production, and altered metabolic states, all of which contribute to a profoundly immunosuppressive microenvironment [6, 7].

**FIGURE 1:**
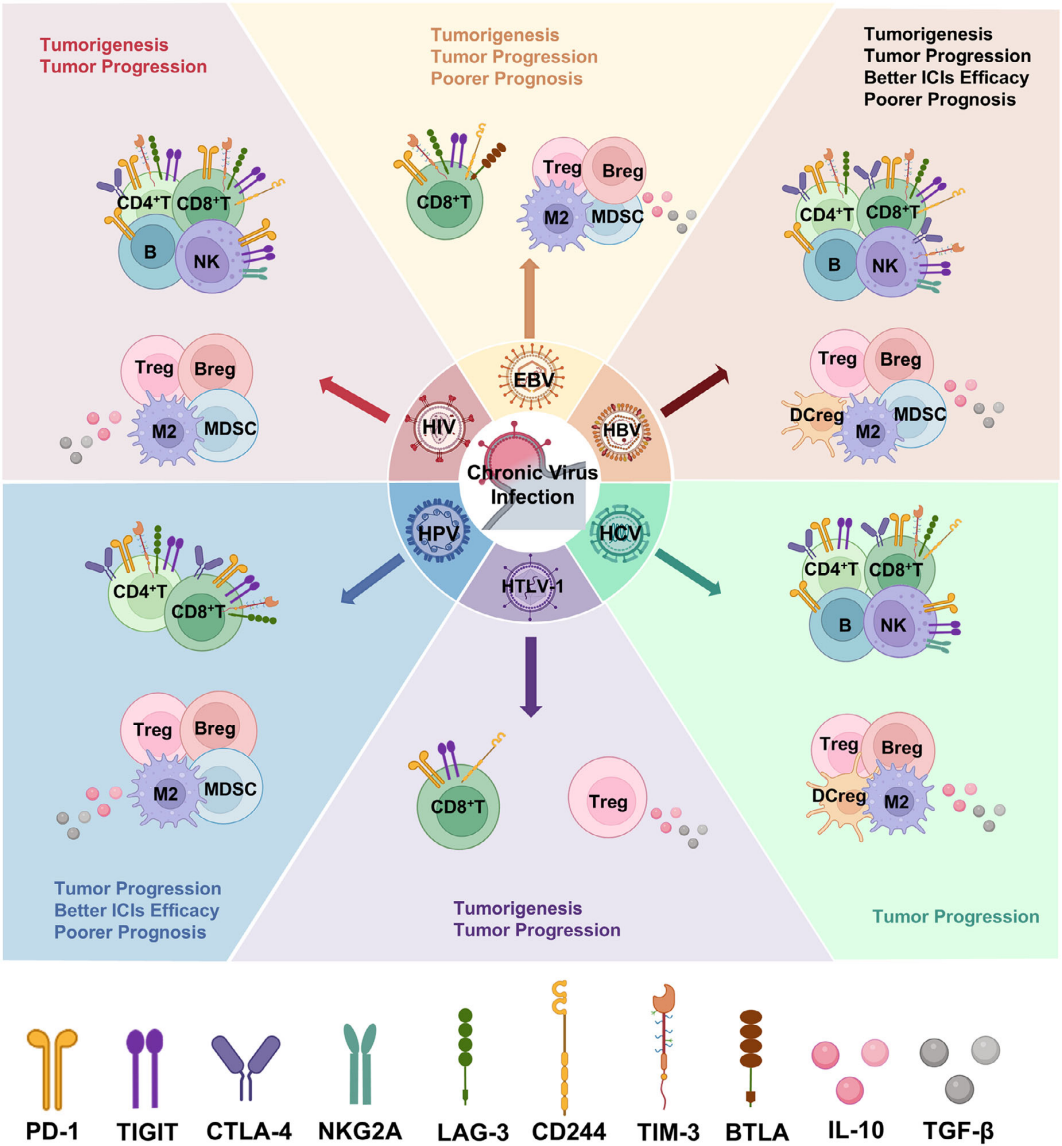
Chronic viral infection drives immunosuppressive reprogramming to fuel tumor pathogenesis. Persistent viral infections induce multilayered immunosuppression through three interconnected mechanisms: (1) immune cell exhaustion—characterized by dysfunctional cytotoxic lymphocytes (e.g., exhausted T/NK/B cells) with elevated checkpoint expression (PD‐1, CTLA‐4, TIGIT); (2) expansion of regulatory cell populations—including MDSCs, Tregs, Bregs, DCregs, and M2 macrophages; (3) inhibitory cytokine storm—sustained release of IL‐10 and TGF‐β. This triad collaboratively cripples tumor immune surveillance, accelerates oncogenesis, and confers resistance to immune checkpoint inhibitors (ICIs). Notably, virus‐specific variations in immunosuppressive pathways (e.g., HBV vs. HPV) differentially shape tumor progression and therapeutic outcomes. Breg, regulatory B cell; BTLA, B‐ and T‐lymphocyte attenuator; CTLA‐4, cytotoxic T lymphocyte antigen‐4; DCreg, regulatory dendritic cell; EBV, Epstein–Barr virus; HBV, hepatitis B virus; HCV, hepatitis C virus; HPV, human papillomavirus; HIV, human immunodeficiency virus; HTLV‐1, human T‐cell leukemia virus type 1; ICIs, immune checkpoint inhibitors; IL‐10, interleukin‐10; LAG‐3, lymphocyte activation gene‐3; MDSC, myeloid‐derived suppressor cell; M2, M2 macrophage; NK, natural killer cell; PD‐1, programmed cell death protein‐1; Treg, regulatory T cell; TIGIT, T cell immunoreceptor with immunoglobulin and ITIM domain; TIM‐3, T cell immunoglobulin and mucin‐domain containing‐3; TGF‐β, transforming growth factor‐β;. (Created with BioRender.com.)

Notably, immune exhaustion driven by chronic infection is correlated with advanced tumor stages, poor response to treatment, and diminished survival across multiple malignancies [8, 9]. This connection underpins our proposal of the chronic infection–exhaustion–tumor axis. Chronic infections arise when pathogens evade immune elimination, forming persistent reservoirs that actively reshape the immune landscape [10]. Through diverse immunomodulatory mechanisms, including sustained antigen exposure, chronic inflammation, and dysregulated immune checkpoint expression, such infections foster an immunosuppressive TME. This reconfigured microenvironment exerts influence over cancer initiation, malignant progression, resistance to therapy, and clinical outcomes [11, 12, 13, 14] (Figure 1). Exhaustion of the immune compartment represents a pivotal component within this process.

This review begins by delineating the molecular drivers and phenotypic hallmarks of immune exhaustion across the contexts of chronic infection and cancer, encompassing T cells, B cells, and NK cells, and provide a comparative analysis between these two conditions. We then examine how chronic viral and bacterial infections promote tumorigenic microenvironments via immune exhaustion, facilitating both tumor development and progression. Finally, we systematically evaluate current therapeutic strategies designed to reverse immune exhaustion, such as immune checkpoint inhibitors (ICIs), with consideration of the distinct features of cancer and chronic infection. By exploring the chronic infection–exhaustion–tumor axis, this work offers new insights spanning virology, immunology, and oncology, and proposes a roadmap for precision interventions aimed at disrupting this pathogenic axis.

## 2 Immune Exhaustion in Chronic Infection

### 2.1 T Cell Exhaustion

T cell exhaustion represents a critical mechanism of immune dysfunction during chronic infection, marked by a gradual deterioration of effector capabilities and persistently overexpressed inhibitory receptors [15]. While the exhaustion process in CD8^+^ T cells has been well delineated, its counterpart in CD4^+^ T cells is less clearly defined [16]. This section systematically reviews the hierarchical process of exhaustion in CD8^+^ T cells, covering mechanisms of development, key characteristics, and phenotypic heterogeneity. A parallel analysis of CD4^+^ T cell exhaustion highlights both shared and distinct regulatory pathways relative to their CD8^+^ counterparts.

#### 2.1.1 CD8^+^ T Cell Exhaustion in Chronic Infection

During acute infection, pathogen‐specific CD8^+^ T cells become activated upon recognizing peptide‐major histocompatibility complex class I (pMHC‐I) complexes presented by antigen‐presenting cells (APCs). Triggered by T cell receptor (TCR) and costimulatory signals, these cells differentiate into effector populations capable of secreting cytolytic mediators (e.g., granzymes and perforins) and inflammatory cytokines such as tumor necrosis factor alpha (TNF‐α) and interferon gamma (IFN‐γ), thereby enabling targeted clearance of pathogens [17]. Following pathogen resolution, most effector cells are eliminated via apoptosis., whereas a distinct subset transitions into a persistent memory pool that mounts rapid responses upon re‐exposure [18].

In contrast, chronic infection leads to persistent antigen exposure. This relentlessly drives pathogen‐specific CD8^+^ T cells down a divergent path, culminating in a state of exhaustion. This exhausted state, initially described in the lymphocytic choriomeningitis virus (LCMV) murine model, is transcriptionally and phenotypically unique, fundamentally distinct from naïve, effector, memory, or anergic T cells [19, 20]. It is marked by numerical decline, diminished cytotoxic activity, and impaired memory formation [21].

Studies indicate that chronic infections establish a microenvironment wherein persistent antigen exposure is the key instigator of CD8^+^ T cell exhaustion, with high antigen loads further reinforcing this dysfunctional state [22]. Key mechanisms include: (1) sustained and intensified TCR signaling [23]; (2) imbalance in cytokine milieu dominated by inhibitory signals such as interleukin‐10 (IL‐10) and transforming growth factor‐beta (TGF‐β) [24]; and (3) immunosuppressive crosstalk via expanded regulatory immune cell populations [25]. For instance, regulatory T cells (Tregs) facilitate the development of exhaustion by secreting immunomodulatory cytokines [3].

Exhausted CD8^+^ T cells undergo a hierarchical loss of effector functions: initially reduced IL‐2 production, followed by impaired cytotoxicity (mediated by granzymes and related enzymes), loss of cytokine polyfunctionality, and diminished proliferative capacity. This decline culminates in attenuated secretion of TNF‐α and IFN‐γ [2]. In late stages, exhausted CD8^+^ T cells may be physically eliminated via Fas/Fas ligand (FasL)‐mediated apoptosis, perforin‐dependent cytotoxicity, or TNF receptor‐related pathways [26].

**FIGURE 2:**
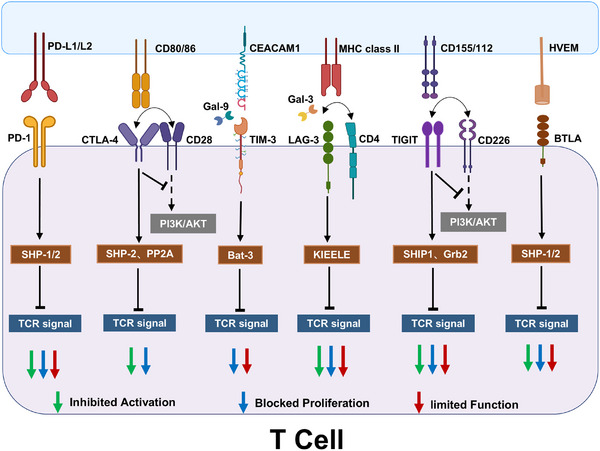
Mechanisms of inhibitory receptor‐mediated T cell suppression. PD‐1: upon ligand binding, recruits SHP‐1/SHP‐2 phosphatases to dephosphorylate TCR signaling components (e.g., Lck and ZAP‐70), thereby blocking TCR activation. CTLA‐4: (1) competes with CD28 for CD80/CD86 binding, suppressing CD28‐dependent PI3K/AKT pathway activation; (2) recruits SHP‐2 and PP2A to attenuate TCR signaling. TIM‐3: ligand engagement (galectin‐9/CEACAM1) induces Bat‐3 release and subsequent TCR signal inhibition. LAG‐3: (1) competes with CD4 for MHC‐II interaction; (2) intracellular KIEELE motif disrupts TCR signaling; (3) binds nonclassical ligands (e.g., galectin‐3) to impair effector function. TIGIT: (1) competes with CD226 for CD155 binding; (2) recruits SHIP1/Grb2 to suppress TCR signaling. BTLA: HVEM binding initiates TCR signaling blockade through phosphatase recruitment. AKT, protein kinase B; CEACAM1, carcinoembryonic antigen‐related cell adhesion molecule 1; Gal‐3, galectin‐3; Gal‐9, galectin‐9; Grb2, growth‐factor‐receptor‐binding protein 2; HVEM, herpesvirus entry mediator; MHC class II, major histocompatibility complex class II; PD‐L1/L2, programmed cell death‐ligand 1/2; PP2A, protein phosphatase 2A; PI3K, phosphatase 3‐kinase; SHIP1, SH2 domain containing inositol‐5‐phosphatase; SHP‐1/2, Src homology domain 2 (SH2)‐containing tyrosine phosphatase‐1/2. (Created with BioRender.com.)

Exhausted CD8^+^ T cells actively and persistently upregulate a broad repertoire of inhibitory receptors. This repertoire includes, but is not limited to, programmed cell death protein‐1 (PD‐1), cytotoxic T‐lymphocyte‐associated protein‐4 (CTLA‐4), T‐cell immunoglobulin and mucin domain‐containing protein 3 (TIM‐3), T‐cell immunoreceptor with immunoglobulin and ITIM domains (TIGIT), lymphocyte activation gene‐3 protein (LAG‐3), and B‐ and T‐lymphocyte attenuator (BTLA). As summarized in Figure 2, these receptors mediate potent suppression of T cell function [27, 28, 29, 30, 31, 32]. Exhausted T cells also display distinct transcriptional and epigenetic profiles accompanied by metabolic alterations [33].

Previous studies have revealed heterogeneity within exhausted CD8^+^ T cells during chronic viral infection, identifying two main subsets: a self‐renewing TCF‐1^hi^ population and a more terminally exhausted TCF‐1^low^ subset derived from it [34, 35]. Emerging evidence supports a hierarchical differentiation model characterized by cellular diversity. Using markers such as CX3C chemokine receptor 1 (CX3CR1), Ly108, CXC chemokine receptor 6 (CXCR6), TCF‐1, and T‐bet, Kasmani et al. proposed a four‐stage developmental hierarchy of exhausted CD8^+^ T cells (Tex) [36]: (1) Ly108^+^CX3CR1^−^TCF‐1^+^CXCR6^+^ Tex: progenitor exhausted T cells (Tex^prog^ or T_pex_), capable of self‐renewal and continuously replenishing the Tex pool; (2) CX3CR1^+^CXCR6^+^ Tex: intermediate exhausted T cells (Tex^int^), a transitional subset; (3)CX3CR1^+^CXCR6^−^T‐bet^+^ Tex: effector‐like exhausted T cells (Tex^eff^), exhibiting relatively enhanced cytotoxicity and contributing to control of chronic infection; (4) Ly108^−^CX3CR1^−^PD‐1^+^ Tex: terminally exhausted T cells (Tex^term^), characterized by high coexpression of multiple inhibitory receptors.

The developmental pathways among these subpopulations remain an active area of research. A currently accepted model incorporates both linear and branched differentiation schemes. When CD4^+^ T cell help is deficient, particularly IL‐21 signaling, Tex^prog^ cells are fated for linear differentiation into Tex^int^ and ultimately Tex^term^. Conversely, sufficient help from CD4^+^ T cells permits Tex^int^ cells to bifurcate: some differentiate into Tex^term^, while others develop into Tex^eff^, thereby improving infection control [37].

#### 2.1.2 CD4^+^ T Cell Exhaustion in Chronic Infection

Although CD4^+^ T cells primarily provide ancillary support during acute infections, they transition to become central regulators in managing chronic infections [38]. While the majority of exhaustion research has focused on CD8^+^ T cells, CD4^+^ T cells also develop an exhausted phenotype under persistent antigen exposure. These cells exhibit functional defects analogous to those seen in exhausted CD8^+^ T cells, including diminished production of cytokines such as IFN‐γ, TNF‐α, and IL‐2. Subsequent investigations have increasingly analyzed the exhaustion of CD4^+^ T cell, with particular emphasis on its commonalities and distinctions relative to exhausted CD8^+^ T cell, most of which were carried out in the chronic LCMV infection model [1].

Exhausted CD4^+^ T cells share key features with their CD8^+^ counterparts, including: (1) reduced production of effector cytokines; (2) elevated PD‐1 expression; and (3) transcriptomic analyses reveal conserved exhaustion signatures between the two subsets, with shared transcription factors, inhibitory receptors, and dysregulated pathways (e.g., proliferation) [39].

Notable distinctive features of CD4^+^ T cell exhaustion include: (1) coinhibitory receptor dynamics: exhausted CD4^+^ T cells display differential expression patterns of inhibitory receptors, with studies showing preferential upregulation of CTLA‐4 and BTLA, in addition to higher PD‐1 expression, compared with exhausted CD8^+^ T cells [39]; (2) functional divergence: exhausted CD4^+^ T cells exhibit elevated production of IL‐10 and IL‐21, suggesting altered functional characteristics and potentially differentiation patterns [40]. Furthermore, a unique predisposition of CD4^+^ T toward T follicular helper cell differentiation trajectories is frequently observed in chronic infections, but whether the latter are derived from exhausted CD4^+^ T cells requires further investigation [41].

### 2.2 NK Cell Exhaustion in Chronic Infection Pathogenesis

NK cells play a crucial role in infection control by serving as a bridge between innate and adaptive immunity [42]. They mediate direct elimination of infected cells in an antigen‐independent manner [43]. Studies of chronic infections, including human papillomavirus (HPV), human immunodeficiency virus (HIV), hepatitis B virus (HBV), and hepatitis C virus (HCV), have shown that pathogens commonly evade NK cell surveillance through shared strategies: (1) reduced cytokine production to suppress NK cell activation and proliferation and (2) downregulation of NK cell activating receptors (e.g., NKp30, NKp46), thereby impairing target recognition and cytotoxicity [44, 45, 46].

Under conditions of chronic antigen exposure, NK cells develop exhaustion‐like phenotypes reminiscent of T cell exhaustion. For instance, during persistent HIV infection, NK cells exhibit elevated expression of inhibitory receptors (e.g., NKG2A) alongside diminished cytotoxic activity and proliferative capacity [6].

### 2.3 B Cell Exhaustion: An Emerging Frontier in Chronic Infection Immunopathology

Although T cell exhaustion remains the central focus of research, and studies on NK cell exhaustion are accumulating, B cells have also been observed to develop exhaustion‐like characteristics in chronic infections. These include: (1) elevated expression of inhibitory receptors such as Fc receptor‐like‐4 (FCRL4), CD22, and PD‐1 [47]. PD‐1 blockade has been shown to partially restore B cells function [48]; (2) altered expression of homing receptors, chemokines, and adhesion molecules [49]; (3) an atypical CD21^−^CD27^−^ surface phenotype [48]; (4) impaired proliferative capacity and compromised effector functions, including constrained immunoglobulin diversity and reduced cytokine secretion [50, 51].

Echoing the characteristics of exhaustion, exhausted B cells demonstrate disrupted signaling pathways, leading to abnormal differentiation, effector functions, and tissue homing [48]. Furthermore, chronic infections drive B cell differentiation into regulatory B cells (Bregs) that secrete IL‐10 to suppress CD4^+^ T cell activity [52].

## 3 Immune Exhaustion in Cancer

### 3.1 T Cell Exhaustion

Although T cell exhaustion was initially defined and studied in the context of chronic infection, similar functional impairments have also been observed in a variety of tumors [53]. Despite both conditions being subjected to chronic antigenic stimulation, the nature of T cell exhaustion differs between the two scenarios. First, infections are systemic, whereas tumors are localized and often multifocal. Second, the TME introduces additional factors, such as nutrient deprivation and the infiltration of suppressive immune cells, which further modulate T cell function. Exhausted T cells contribute to immune evasion and tumor progression, and their accumulation is generally associated with poor clinical outcomes [54].

#### 3.1.1 CD8^+^ T Cell Exhaustion in Cancer: Comparison With Infection Scenarios

The exhaustion of CD8^+^ T cells within tumors is underpinned by multiple mechanisms: (1) persistent tumor antigen stimulation drives the high expression of inhibitory receptors, including PD‐1, CTLA‐4, TIM‐3, LAG‐3, and TIGIT. These immune checkpoints (ICs) suppress CD8^+^ T cell activation, proliferation, survival, and effector functions through downstream signaling pathways [55]; (2) immunosuppressive factors within the TME. Various immune suppressor cells, such as Tregs, Bregs, myeloid‐derived suppressor cell (MDSCs), and M2 macrophages, engage in crosstalk with CD8^+^ T cells. The secretion of immunosuppressive cytokines (IL‐10 and TGF‐β) by these cells further inhibits T cell function [56, 57]; (3) metabolic characteristics of the TME, including hypoxia, low pH, accumulation of metabolic waste products, and nutrient deprivation, affect T cell bioenergetics. For instance, adenosine impairs the cytotoxic activity and proliferative capacity of CD8^+^ T cells [58]; (4) the extracellular matrix (ECM) serves as a mechanical barrier, collaborating with fibroblasts to hinder T cell infiltration and activity [59].

**FIGURE 3:**
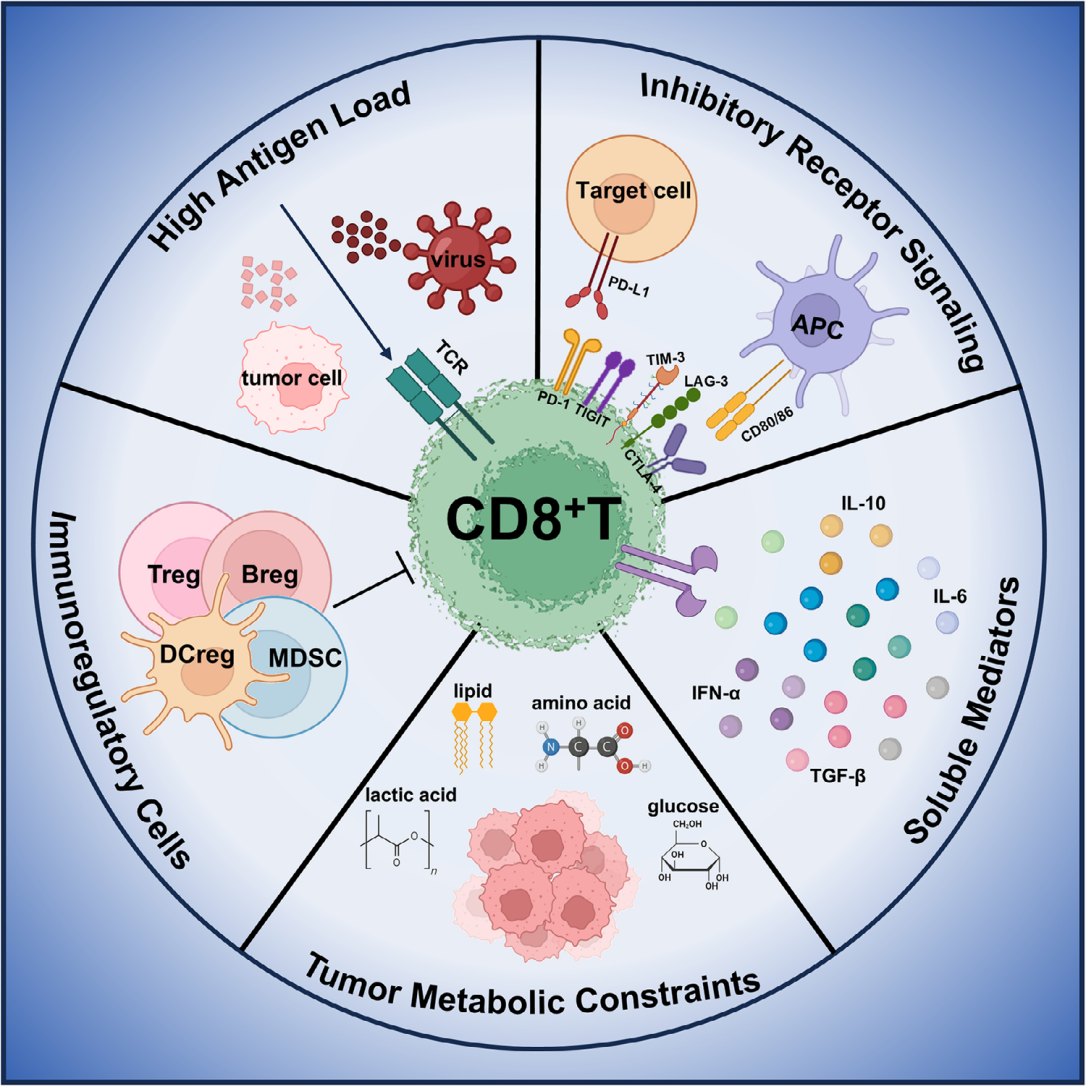
Mechanisms driving CD8^+^ T cell exhaustion. (1) High antigen burden. Sustained antigen exposure constitutes the primary driver of T cell exhaustion, where both antigen magnitude and persistence duration critically determine exhaustion progression. (2) Inhibitory receptor signaling. Upregulated inhibitory receptors on exhausted T cells engage cognate ligands expressed by APCs and target cells, initiating suppressive signaling cascades that dampen T cell functionality. (3) Soluble mediators. Proinflammatory cytokines synergize with regulatory cytokines to promote exhaustion, with inflammatory mediators frequently acting as intermediaries between inflammatory signaling and other exhaustion‐inducing pathways. (4) Immunosuppressive cell populations. Regulatory immune cells actively impair CD8^+^ T cell activation, differentiation, and effector functions. Compromised CD4^+^ T cell support further exacerbates the exhaustion process. (5) Tumor metabolic constraints. Metabolic competition within the tumor microenvironment deprives CD8^+^ T cells of essential nutrients. Hypoxic conditions and cytotoxic byproducts from heightened tumor metabolism collectively impair T cell differentiation/function while accelerating exhaustion progression. APC, antigen‐presenting cell; IFN‐α/β, interferon‐α/β; IL‐6, interleukin‐6; TCR, T cell receptor. (Created with BioRender.com.)

Under these stimuli, tumor‐infiltrating exhausted CD8^+^ T cells exhibit hallmark features of exhaustion: impaired proliferation, reduced cytokine production, elevated expression of inhibitory receptors, and loss of cytotoxicity, similar to what is observed in chronic infection [60]. They also display distinct metabolic, transcriptional, and epigenetic alterations that stabilize the exhausted phenotype [55]. The mechanisms of CD8^+^ T cell exhaustion in chronic infection and tumor are summarized in Figure 3.

It is crucial to recognize that the developmental trajectories of exhausted CD8^+^ T cells diverge between chronic infection and tumor. In chronic infection, naive T cells encounter antigens acutely in an inflammatory environment, differentiating into effector cells and then progressively losing their effector functions to become Tex [61]. In contrast, during tumorigenesis, tumor antigens are presented in a noninflammatory environment, leading to lower levels of TCR stimulation. This results in the formation of memory cells. Under continuous TCR stimulation, these memory cells further differentiate into Tex. However, whether these memory cells undergo an effector phase and whether all Tex cells originate from memory cells remains an unresolved issue [62].

**FIGURE 4:**
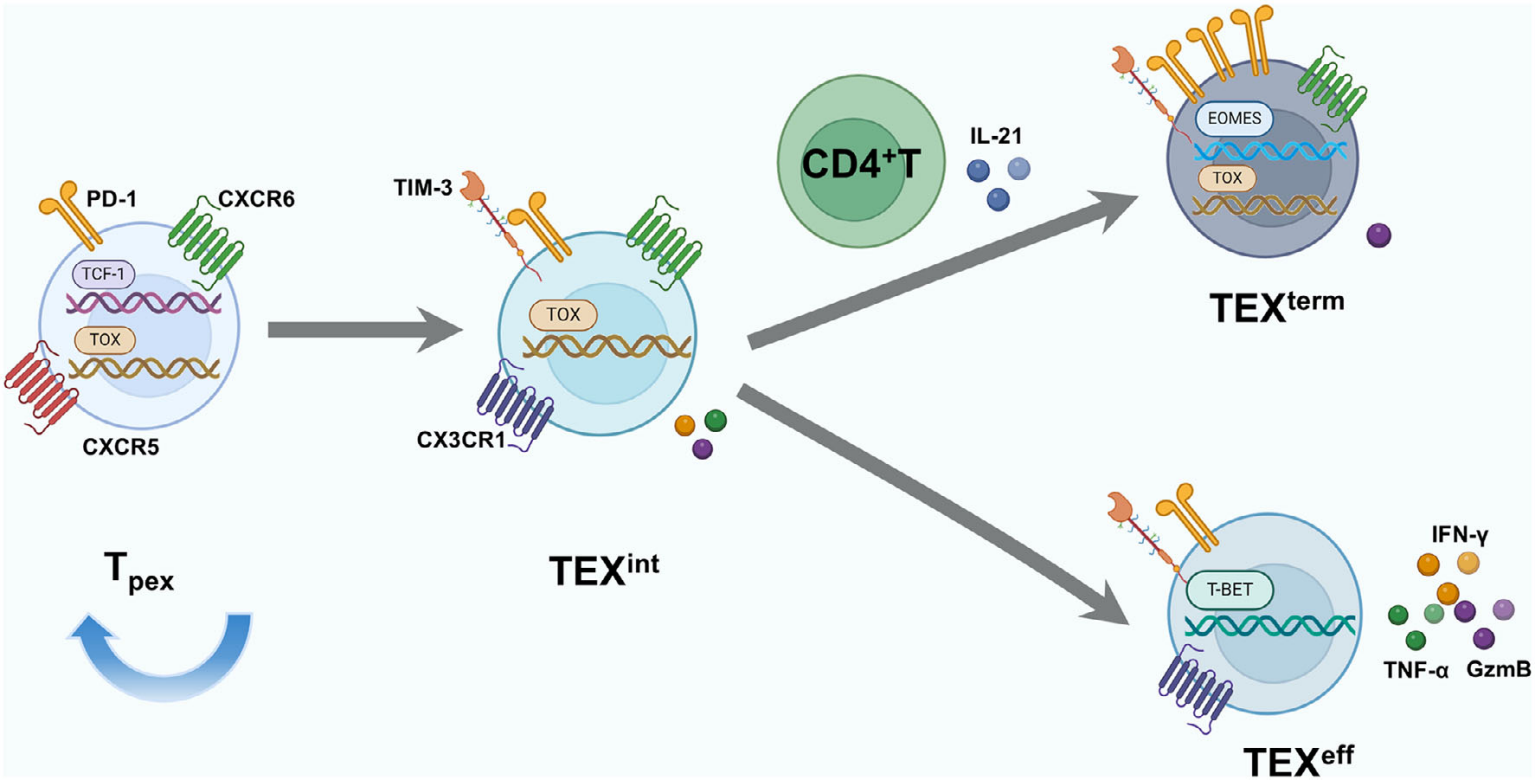
Developmental trajectories of CD8^+^ T cell exhaustion. T_pex_ cells possess stem cell‐like properties and serve as the self‐renewing reservoir for the exhausted T cell pool. Their differentiation proceeds through two distinct pathways: (1) in the absence of CD4^+^ T cell help and IL‐21 signaling, T_pex_ → TEX^int^ → TEX^term^ without generating TEX^eff^; (2) when supported by CD4^+^ T cells and IL‐21, TEX^int^ cells bifurcate into either terminally differentiated TEX^term^ or effector‐competent TEX^eff^. Notably, functional heterogeneity exists within the T_pex_ compartment: CD69^−^Ly108^+^ and CD62L^+^ T_pex_ subsets demonstrate enhanced proliferative capacity and superior responsiveness to immune checkpoint inhibitors (ICIs). CXCR5, CXC chemokine receptor 5; CXCR6, CXC chemokine receptor 6; CX3CR1, CX3C chemokine receptor 1; Eomes, eomesodermin; GzmB, granzyme B; IRs, inhibitory receptors; T_pex_, progenitor exhausted CD8^+^ T; TEX^int^, intermediate exhausted CD8^+^ T; TEX^term^, terminally exhausted CD8^+^ T; TEX^eff^, effector‐like exhausted CD8^+^ T; TCF‐1, T‐cell factor 1; TOX, Thymocyte selection‐associated high mobility group box protein; T‐bet, T‐box expressed in T cell. (Created with BioRender.com.)

The hierarchical evolution of Tex in tumors is less clearly defined than in infection, partly due to the multisite nature of antitumor responses [63]. Current evidence supports a differentiation process from T_pex_ to Tex (Figure 4). T_pex_ predominantly reside within tumor‐draining lymph nodes, exhibiting stem cell‐like properties of self‐renewal and differentiation, thereby replenishing both intratumoral T_pex_ and Tex pools [64, 65, 66]. They are the primary responders to ICIs and correlate with favorable treatment outcomes [67, 68, 69]. Tex^int^ retain proliferative capacity and exhibit robust responses to ICIs. Recent studies have demonstrated substantial expansion of Tex^int^ cells in the splenic white pulp following ICIs treatment [70]. In contrast, Tex^term^ characterized by high expression of TOX and multiple inhibitory receptors have been shown to be resistant to ICIs [67]. Given the differential responsiveness of Tex subpopulations to ICIs, further research is required to elucidate the precise differentiation trajectories of Tex cells in tumors. Integrating epigenetic and transcriptomic approaches may offer clearer insights into these processes.

#### 3.1.2 CD4^+^ T Cell Exhaustion in Cancer: An Underexplored Frontier

CD4^+^ T cells mediate antitumor immunity primarily by providing collaborative support to other immune cells, but they also possess direct or indirect cytotoxicity toward tumor cells [1]. Accumulating evidence indicates that the presence of exhausted CD4^+^ T cells within tumors correlates with unfavorable clinical outcomes. In leukemia, accumulation of TIM‐3^+^ exhausted CD4^+^ T cells correlates with increased relapse risk [71]. In non‐small cell lung cancer (NSCLC), high PD‐1 and LAG‐3 expression on peripheral CD4^+^ T cells is linked to treatment resistance and immunosuppression [72].

Exhausted CD4^+^ T cells share transcriptional and phenotypic features with exhausted CD8^+^ T cells, including upregulation of inhibitory receptors and expression of exhaustion‐related transcription factors like TOX [73]. In mouse models, exhausted CD4^+^ T cells resemble terminally exhausted CD8^+^ T cells, with elevated PD‐1, TIM‐3, and IFN‐γ, but reduced TCF‐1 [74]. In breast cancer patients, CD39^+^ conventional CD4^+^ T cells exhibit higher expression of inhibitory receptors, including PD‐1, TIGIT, TIM‐3, LAG‐3, and CD244, along with increased TOX, T‐bet, and Eomes, alongside a reduction in TCF‐1 expression, mirroring the differentiation trajectory from Tex^prog^ to Tex^int^ seen in CD8^+^ T cells. This suggests that the exhaustion program of CD4^+^ T cells may follow a similar trajectory to that of CD8^+^ T cells [75].

Several aspects of CD4^+^ exhaustion are unique. First, animal studies in oral squamous cell carcinoma have shown that exhausted CD4^+^ T cells appear earlier than exhausted CD8^+^ T cells [76]. Second, despite sharing phenotypic and transcriptional similarities with terminally exhausted CD8^+^ T cells, CD4^+^ T cells retain responsiveness to PD‐1 blockade [74, 77]. Last, the remarkable capacity of CD4^+^ T cells to differentiate into highly specialized subsets, the generalization of common features across exhausted CD4^+^ T cells is inherently difficult. Consequently, it remains challenging to fully comprehend their impact on tumors and to appropriately adjust immunotherapy strategies.

There is a paucity of both basic and clinical evidence elucidating the driving factors of CD4^+^ T cell exhaustion in tumors. Nevertheless, based on current understanding, the exhausted state in CD4^+^ T cells is considered to be driven by the elevated expression of inhibitory receptors, chronic antigen stimulation, and an immunosuppressive cytokine milieu [55]. Additionally, amino acids influence T cell survival, activation, differentiation, and function, implicated in the exacerbation of CD4^+^ T cell exhaustion [78]. In melanoma, the tryptophan metabolite kynurenine suppresses CD4^+^ T cell activation, thereby contributing to their exhaustion [79]. In tumor‐bearing mice, cancer cell‐mediated methionine deprivation upregulates PD‐1 expression on CD4^+^ T cells, resulting in T cell exhaustion [80].

Indeed, compared with CD8^+^ T cells, our current understanding of CD4^+^ T cell exhaustion remains anemic, underscoring the critical need for further research into the transcriptional, metabolic, and epigenetic dynamics of this process [81]. A key uncertainty is whether its exhaustion progresses in a similarly stage‐ and lineage‐dependent manner. To more effectively target exhausted CD4^+^ T cells for tumor immunotherapy, it is essential to continue exploring the underlying mechanisms of CD4^+^ T cell exhaustion, with a particular focus on precisely characterizing the exhaustion state and effects of each subset during tumor progression.

### 3.2 NK Cell Exhaustion

NK cells controlling tumor progression through three primary mechanisms: (1) direct cytotoxicity against cancer cells via FasL, TNF‐related apoptosis inducing ligand (TRAIL), and cytotoxic granules (perforin, granzymes); (2) antibody‐dependent cellular cytotoxicity (ADCC), leading to the destruction of tumor cells; and (3) crosstalk with other immune cells via the secretion of cytokines and chemokines, which bolster the overall antitumor response. However, NK cells can undergo exhaustion in tumors, with diminished cytotoxic activity. Exhausted NK cells contribute to tumor progression and metastasis and are strongly linked to poor prognosis in cancer patients [82, 83]. Current research efforts are dedicated to uncovering the characteristics and mechanisms underlying NK cell exhaustion in tumors.

#### 3.2.1 Characteristics of NK Cell Exhaustion

NK cell exhaustion in the tumor milieu is defined by a constellation of deficits: impaired proliferation, diminished cytotoxicity (with reduced secretion of perforin and granzyme), decreased cytokine production (such as IFN‐γ and TNF‐α), upregulation of inhibitory receptors (e.g., NKG2A), downregulation of activating receptors (e.g., NKG2D), metabolic dysfunction, and altered expression of key transcription factors like T‐bet [84, 85].

NK cells comprise two principal subpopulations: CD56^bright^, which primarily secretes proinflammatory cytokines, and CD56^dim^, a more mature subset with stronger cytotoxicity [86]. In acute myeloid leukemia (AML), CD56^dim^ NK cells manifest features of exhaustion. These hallmarks encompass defective proliferation and activation, upregulation of inhibitory receptors (e.g., PD‐1), and reduced IFN‐γ production [87].

#### 3.2.2 NK Cell Exhaustion Pathways

Several fundamental drivers underlie NK cell exhaustion within the TME: (1) *Signaling imbalance*. This imbalance stems from the dysregulated surface density of activating versus inhibitory receptors on NK cells. These receptors critically govern cytotoxic functions, including degranulation and cytokine production [88]. As exemplified in colorectal cancer (CRC) patients, TIGIT overexpression on the NK cell surface suppresses the production of IFN‐γ and TRAIL [89]. Furthermore, NKG2D downregulation, observed across multiple cancer types, results in diminished NK cell activity [84]. (2) *Inhibitory effects of tumor‐secreted factors*. Soluble mediators, cytokines, exosomes, and other factors secreted by tumor cells can suppress NK cell activation, cytotoxicity, and cytokine production. For instance, tumor cells secrete prostaglandin E2, which downregulates activating receptors on NK cells and inhibits IFN‐γ production [90, 91]. TGF‐β can alter the balance of activating and inhibitory receptors on NK cells, leading to NK cell exhaustion [92]. (3) *Regulation by immunosuppressive cells*. Various immunosuppressive cells in the TME, including Tregs, MDSCs, tumor‐associated macrophages (TAMs), and neutrophils, collectively suppress the effector functions of NK cells. TAMs secrete chemokines that recruit Tregs to the TME, where they release TGF‐β suppressing NK cell proliferation and cytotoxicity. (4) *Metabolic damage from the TME*. Similar to their effects on T cells, the unique metabolic characteristics of the TME impair NK cell proliferation, survival, and cytotoxicity. For instance, in multiple myeloma, hypoxia compromises the ability of NK cells to generate perforin and granzyme B [93].

#### 3.2.3 Inhibitory Receptors on Exhausted NK Cell

PD‐1, LAG‐3, TIM‐3, TIGIT, CD96, and NKG2A are among the inhibitory receptors implicated in NK cell exhaustion. PD‐1 assumes a dual function, participating in both the activation and exhaustion of NK cells. This complexity accounts for the conflicting reports regarding PD‐1 levels on exhausted cells [94]. For instance, in gastrointestinal cancer patients, upregulation of PD‐1 on exhausted NK cells promotes disease progression [83]. In contrast, melanoma studies found comparable PD‐1 levels in patients and healthy individuals. Instead, TIM‐3 levels on peripheral NK cells showed association with tumor staging [95]. Despite constitutive expression on mature NK cells, TIM‐3 interaction with tumor ligands promotes exhaustion [96]. TIGIT represents a more pivotal exhaustion marker of NK cell. Zhang et al. reported that in colon cancer patients, tumor‐infiltrating NK cells showed minimal expression of PD‐1 and CTLA‐4, but had elevated levels of TIGIT. Moreover, blocking TIGIT in tumor‐bearing mice prevented NK cell exhaustion and restored their antitumor activity [89]. In hepatocellular carcinoma (HCC), exhausted CD96^+^ NK cell exhaustion with impaired cytokine production and cytotoxicity is associated with poor clinical outcome [97]. NKG2A represents a critical checkpoint in NK cells, exacerbating their exhaustion within the AML context [98].

### 3.3 B Cell Exhaustion

Within tumors, persistent antigen stimulation coupled with elevated expression of inhibitory checkpoint molecules drives B cells into an exhausted state. This dysfunctional condition manifests in diminished cytokine and antibody production, low metabolic activity, and impaired antigen processing and presentation [55, 99]. Consequently, exhausted B cells impair humoral immune responses, facilitate tumor immune escape and contribute to inferior clinical outcomes. In CRC patients, BTLA^+^ B cells are correlated with shortened survival, supporting this notion [100]. Nevertheless, the precise mechanistic underpinnings of B cell exhaustion in malignancies await further elucidation.

## 4 The Impact of Chronic Infection‐Induced Immune Exhaustion on Tumor Immunosurveillance

### 4.1 Chronic Virus Infection

Most viral infections are self‐limiting, clearing within months and pose no carcinogenic risk. Chronic infection arises when pathogens evade immune clearance, establishing persistent reservoirs that actively sculpt the immune landscape [10]. Through multifaceted immunomodulatory mechanisms, including sustained antigen stimulation, chronic inflammation, and dysregulated cytokine networks, these infections drive immunosuppressive TME, thereby influencing tumor initiation, progression, therapeutic resistance, and clinical outcomes [11, 12, 13, 14]. This section delineates how chronic infections with HBV, HCV, HPV, HIV, Epstein–Barr virus (EBV) and human T‐cell leukemia virus type 1 (HTLV‐1), which are established oncogenic pathogens, drive tumorigenesis and progression through immune exhaustion mechanisms, with Table 1 encapsulating their convergent immunosuppressive TME signatures.

**TABLE 1:** Immune microenvironment alterations in chronic viral infections.

Virus	Manifestations	References
HBV	Immune cell exhaustion (CD8^+^ T, CD4^+^ T, NK, B)	[48, 101, 102, 103]
	Increased immunosuppressive cells (Tregs, Bregs, MDSCs, DCregs, M2 macrophages)	[11, 104, 105, 106, 107]
	Elevated IL‐10 and TGF‐β production	[11]
	Upregulated coinhibitory receptors (CD8 ^+^T: PD‐1, TIM‐3, TIGIT, LAG‐3, CTLA‐4, CD244 CD4^+^T: PD‐1, TIM‐3, LAG‐3, CTLA‐4, BTLA)	[108, 109, 110, 111, 112, 113] [102, 114, 115, 116]
	Expansion of CD56^−^ NK cell subset	[117]
HCV	Immune cell exhaustion (CD8^+^ T, CD4^+^ T, NK, B)	[118, 119, 120, 121]
	Increased immunosuppressive cells (Tregs, Bregs, DCregs, M2 macrophages)	[122, 123, 124, 125]
	Elevated IL‐10 and TGF‐β production	[126]
	Upregulated coinhibitory receptors (PD‐1, TIM‐3, CTLA‐4, LAG‐3, CD244, BTLA)	[127]
	Expansion of CD56^−^ NK cell subset	[128]
HPV	Immune cell exhaustion (CD8^+^ T, CD4^+^ T, NK)	[129, 130, 131]
	Increased immunosuppressive cells (Tregs, Bregs, MDSCs, M2 macrophages)	[129, 132, 133]
	Elevated IL‐10 and TGF‐β production	[134]
	Upregulated coinhibitory receptors (PD‐1, CTLA‐4, LAG‐3, TIM‐3)	[135]
HIV	Immune cell exhaustion (CD8^+^ T, CD4^+^ T, NK, B)	[51, 136, 137]
	Increased immunosuppressive cells (Tregs, Bregs, MDSCs, M2 macrophages)	[138, 139, 140, 141]
	Elevated IL‐10 and TGF‐β production	[142, 143]
	Upregulated coinhibitory receptors (CD8^+^T: PD‐1, TIM‐3, LAG‐3, TIGIT CD4^+^T: PD‐1, CTLA‐4, TIM‐3, LAG‐3, TIGIT)	[144, 145, 146, 147] [148, 149, 150]
	Expansion of CD56^−^ NK cell subset	[128]
EBV	Immune cell exhaustion (CD8^+^ T, CD4^+^ T, NK)	[151, 152]
	Increased immunosuppressive cells (Tregs, MDSCs, Bregs, M2 macrophages)	[153, 154, 155]
	Elevated IL‐10 and TGF‐β production	[156]
	Upregulated coinhibitory receptors (PD‐1, CTLA‐4, TIM‐3, LAG‐3, CD244, BTLA, TIGIT)	[157, 158]
	EBV‐infected B cells acquire Breg‐like phenotype	[159]
	Expansion of CD56^−^ NK cell subset	[160]
HTLV**‐1**	Immune cell exhaustion (CD8^+^ T)	[161]
	Elevated IL‐10 and TGF‐β production	[162]
	Upregulated coinhibitory receptors (PD‐1, TIGIT, CD244)	[161, 163, 164]
	HTLV‐1‐infected CD4^+^ T cells acquire Treg‐like function	[165]

*Abbreviations*: Bregs, regulatory B cells; BTLA, B‑ and T‑lymphocyte attenuator; CD244, natural killer cell receptor 2B4; CTLA‑4, cytotoxic T‑lymphocyte‑associated protein 4; DCregs, regulatory dendritic cells; EBV, Epstein–Barr virus; HBV, hepatitis B virus; HCV, hepatitis C virus; HIV, human immunodeficiency virus; HPV, human papillomavirus; HTLV‑1, human T‑cell leukemia virus type 1; IL‑10, interleukin‑10; LAG‑3, lymphocyte activation gene‑3; MDSCs, myeloid‑derived suppressor cells; PD‑1, programmed cell death protein 1; TGF‑β, transforming growth factor‑beta; TIGIT, T‑cell immunoreceptor with Ig and ITIM domains; TIM‑3, T‑cell immunoglobulin and mucin‑domain containing‑3; Tregs, regulatory T cells.

#### 4.1.1 HBV‐Induced Immunological Remodeling in Hepatocarcinogenesis

The foremost risk factor for HCC is persistent HBV infection [166]. In chronic HBV infection, immune cells become exhausted, with overexpression of various inhibitory receptors and impaired effector function. This results in an immunosuppressive microenvironment that promotes tumorigenesis and tumor progression. Studies show that the TME in HBV‐positive HCC exhibits stronger immunosuppression and exhaustion compared with HBV‐negative HCC [14]. In HBV‐related HCC, exhausted CD8^+^ T cells with elevated PD‐1 and TIGIT levels are significantly increased and implicated in fostering tumor recurrence and progression [116]. Coexpression of TIGIT and TIM‐3 on exhausted NK cells is tightly linked to disease progression and unfavorable outcomes in HBV–HCC patients [166]. Additionally, HBV‐positive HCC patients tend to respond more favorably to ICIs than those without HBV infection. This therapeutic advantage appears to correlate with the upregulation of inhibitory receptors (e.g., PD‐1/BTLA on CD4^+^ T cells and PD‐1/TIGIT on CD8^+^ T cells). Conversely, responses to tyrosine kinase inhibitors are comparable across both cohorts [167]. These findings highlight the considerable impact of chronic HBV infection on shaping immunotherapy efficacy and clinical prognosis.

#### 4.1.2 HCV: Prominent Role of Immune Checkpoints

HCV infection constitutes another significant etiological factor for liver cancer [168]. Continuous stimulation by hepatitis C antigens leads to exhausted HCV‐specific CD8^+^ T cells, expressed inhibitory receptors include significantly elevated PD‐1, CD244, BTLA, and TIM‐3, as well as moderately elevated CTLA‐4 and LAG‐3 levels [127]. A decline in CD4^+^ T cell count occurs alongside diminished secretion of key cytokines including IL‐2 [169]. Persistent HCV infection also drives exhaustion in NK and B cell populations [119, 170]. The amplification of immunosuppressive cells and inhibitory cytokines, combined with immune exhaustion, forms an inhibitory TME that impairs the antitumor immune response and promotes tumor progression in HCV‐positive HCC patients. Clinical trials have shown that HCV‐related HCC patients respond more effectively to ICIs than nonvirus‐related HCC patients, possibly attributed to the elevated expression of various inhibitory receptors within the TME [167].

#### 4.1.3 HPV: A Dichotomy of Exhaustion and Response

Chronic HPV infection gradually shapes the immune microenvironment, exacerbating its inhibitory effects during tumor progression [171]. Compared with HPV‐negative tumors, HPV‐positive penile squamous cell carcinoma shows an increase in M2‐like macrophages, Tregs, and exhausted CD8^+^ T cells, possibly partly driven by the persistent antigen stimulation from the HPV virus [172]. In HPV‐positive head and neck squamous cell carcinoma (HNSCC), a higher number of exhausted CD8^+^ T cells (PD‐1^+^ or PD‐1^+^LAG‐3^+^) have also been detected, highlighting the association between HPV infection and T cell exhaustion [173]. An imbalance between Tregs and CD4^+^ T cells was revealed during the progression from persistent HPV infection to precancerous lesions, promoting carcinogenesis [129]. In HPV‐positive cervical intraepithelial neoplasia (CIN) patients, elevated expression of NK cell inhibitory receptors TIGIT and killer cell lectin‐like receptor subfamily G member 1 (KLRG1) and reduced cytokine secretion suggest a potential exhausted state. The number of these cells correlates with increasing CIN grade, indicating their involvement in tumor progression [131]. Chronic HPV infection can also impact the efficacy of tumor immunotherapy. Compared with HPV‐negative HNSCC patients, HPV‐positive HNSCC patients exhibit better tumor immunotherapy efficacy, potentially due to the high level and activity of PD‐1 and CTLA‐4 [174]. Studies suggest that CD161‐marked CD8^+^ T cell subsets, which have an exhausted phenotype but retain immunoreactivity, are key contributors to the improved immunotherapy response in HPV‐positive oropharyngeal squamous cell carcinoma patients [175].

#### 4.1.4 HIV: Prototypic Exhaustion Hierarchy

The most prominent feature of chronic HIV infection is impaired immune function, coupled with a high cancer incidence in these patients. This suggests that chronic HIV infection‐mediated immunosuppression plays a role in tumorigenesis [12]. The incidence of non‐Hodgkin lymphoma (NHL) is elevated among persons living with HIV, partly due to immune exhaustion driven by chronic viral antigen stimulation [136]. Lucar et al. found that in chronic HIV infection, long‐term exposure to corresponding ligands downregulates NK cell activating receptors, reducing their tumor‐clearing ability and thereby promoting oncogenesis [176]. Furthermore, a year before cancer diagnosis, individuals with HIV subsequently diagnosed with malignancy show a higher percentage of highly exhausted CD8^+^ T cells than their noncancer counterparts, indicating that such exhaustion mediates the increased oncogenic risk in chronic HIV infection [12].

#### 4.1.5 EBV: A Paradigm of Viral‐Driven Exhaustion and Tolerance

Chronic EBV infection creates a more tolerant immune microenvironment, associated with various types of cancer [177]. Relative to their EBV‐negative counterparts, NHL tumors harboring EBV show heightened PD‐1 and TIGIT levels on CD8^+^ T cells and greater Treg infiltration [158]. Owing to sustained antigen exposure, chronic EBV infection upregulates multiple inhibitory receptors (PD‐1, LAG‐3, TIM‐3, CTLA‐4), thereby inducing exhaustion in T and NK cells [152]. Chronic EBV infection also polarizes macrophages to the M2 type, which promotes T cell exhaustion and tumor progression [178, 179]. Within the context of persistent EBV infection, Bregs have been shown to inhibit T cell proliferation and promote Treg expansion, thereby promoting oncogenesis [159]. This immunosuppressive network is further strengthened by the accumulation of both MDSCs and Tregs, which suppress T cell function and correlate with poor prognosis in EBV‐associated tumors [153, 154]. While EBV‐positive HCC exhibits a denser lymphocytic infiltrate than its EBV‐negative counterpart, the prevailing exhausted CD8^+^ T cells leads to adverse outcomes [180].

#### 4.1.6 HTLV‐1: Retroviral Immune Engineering

HTLV‐1 establishes a persistent, decades‐long latent infection, with roughly 3% of carriers ultimately progressing to adult T‐cell leukemia [181]. The virus facilitates immunosuppression through the upregulation of T cell inhibitory receptors and recruitment of Tregs [182, 183]. HTLV‐1 infection also attenuates innate immunity, leading to impaired NK cell killing, reduced monocyte clearance, and dysfunctional cytokine production by dendritic cells (DCs) [184]. Phenotypically, exhausted CD8^+^ T cells exhibit heightened expression of PD‐1, TIGIT, and CD244 [161, 163, 164]. This immunosuppressive milieu is further reinforced by the concurrent overproduction of IL‐10 and TGF‐β [162]. Notably, HTLV‐1 reprograms infected CD4^+^ T cells to acquire Treg‐like functions, leading to immunodeficiency and subsequent deterioration of diseases, including the development of tumors [165].

### 4.2 Chronic Nonviral Infection

In virus‐associated tumors, pathogens reshape the immune microenvironment during chronic infection, inducing immunosuppression that causes persistent inflammation and impaired immune surveillance, ultimately facilitating cancer development. Indeed, other pathogens can also establish chronic infections that lead to immune exhaustion. In tuberculosis patients, persistent *Mycobacterium tuberculosis* (Mtb) infection drives T cell exhaustion, a state marked by compromised function, diminished secretion of IFN‐γ, TNF‐α, and IL‐2, elevated expression of PD‐1, TIM‐3, and LAG‐3, and metabolic dysregulation [185, 186]. Similarly, chronic infection by the parasitic worm *Echinococcus multilocularis* causes alveolar echinococcosis (AE), wherein exhausted NK cells exhibit elevated TIGIT expression alongside diminished cytotoxicity and cytokine release, a phenotype restored by TIGIT blockade [187]. Collectively, these cases demonstrate that chronic nonviral infections can create an immunosuppressive environment, thereby fostering the development and progression of associated cancers.

#### 4.2.1 Helicobacter Pylori (H. Pylori)

*Helicobacter pylori* (*H. pylori*) infection is established as the predominant risk factor for gastric cancer [188]. Chronic *H. pylori* infection fosters an immunosuppressive microenvironment, characterized by an enrichment of M2 macrophages, Tregs, MDSCs, and regulatory DCs (DCregs), concurrent with T cell exhaustion [189]. The functional impairment of exhausted CD8^+^ T cells compromises immune surveillance, thereby promoting both tumorigenesis and poor prognosis in gastrointestinal cancers [190]. Eradication of *H. pylori* can overcome immune exhaustion, preventing the metachronous recurrence of gastric tumors [191].

#### 4.2.2 Fusobacterium Nucleatum

The gut bacterium *Fusobacterium nucleatum* (FN) is established as an oncogenic contributor to CRC and linked to adverse patient outcomes [192, 193]. This association is related to distinct immune alterations: FN‐positive tumors exhibit increased Tregs than their FN‐negative counterparts, and upregulated PD‐1, TIM‐3, and TIGIT on CD8^+^ T cells under high FN load. These findings suggest that FN infection could promote T cell exhaustion as the tumor advances, contributing to a more profoundly immunosuppressive microenvironment [194].

#### 4.2.3 Staphylococcus Aureus

Approximately 40% of patients with untreated chronic lymphocytic leukemia (CLL) show colonization of the upper respiratory tract by *Staphylococcus aureus* (SA), a condition linked to diminished survival and elevated PD‐1 expression [195]. To explore the impact of chronic SA infection on CLL, S. aureus superantigen was used for chronic stimulation of CLL patient cultures in vitro. This resulted in increased tendency of T cells to acquire an exhausted phenotype, which may contribute to tumor development [195].

## 5 Therapeutic Strategies Targeting Immune Cell Exhaustion in Cancer

**FIGURE 5:**
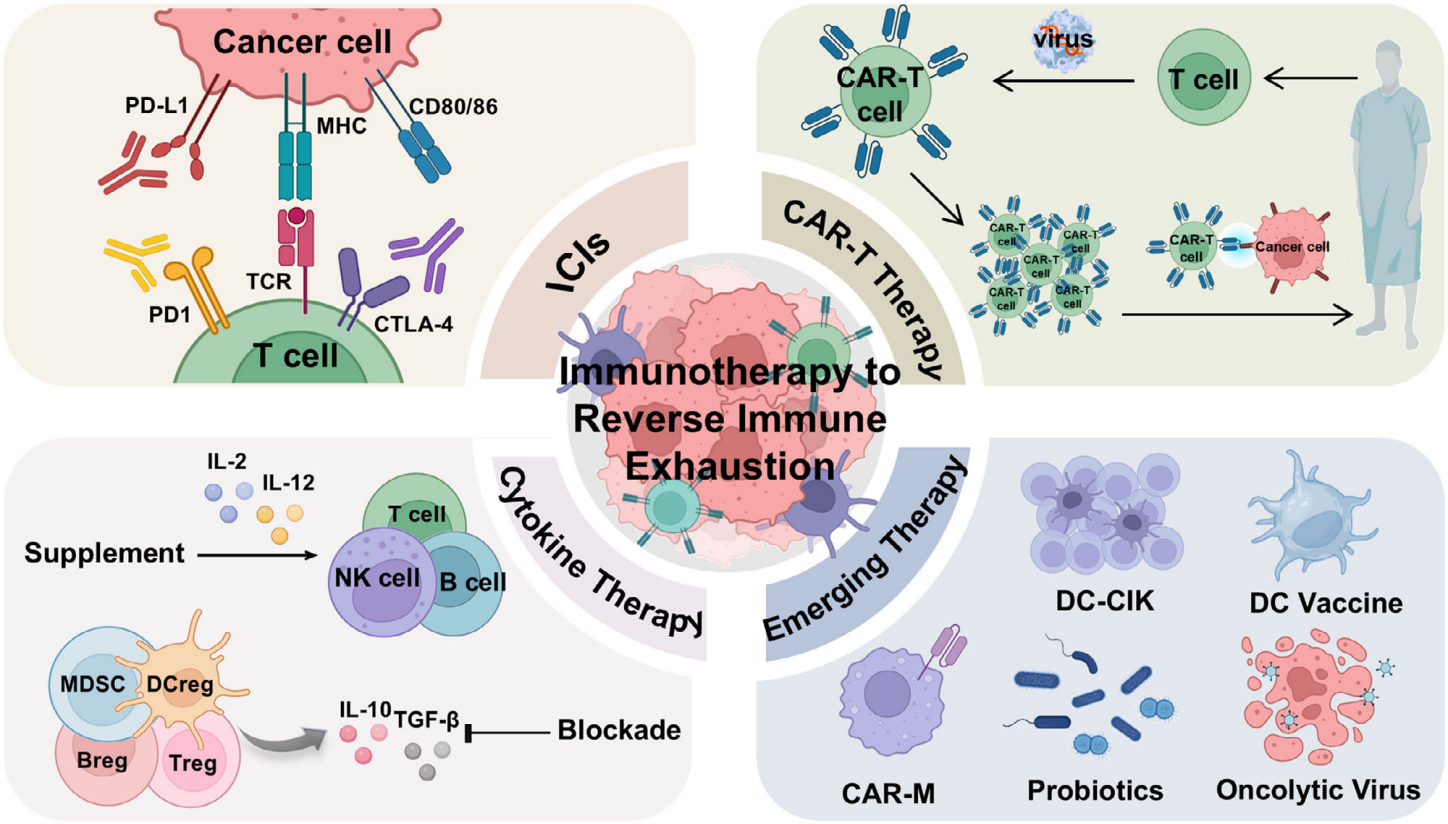
Strategies to reverse immune exhaustion. Currently, there are three primary types of immunotherapies aimed at reversing the exhausted microenvironment: (1) targeting highly expressed inhibitory receptors with ICIs; (2) restoring T‐Cell Function with CAR‐T Therapy; (3) regulating cytokine balance. Additionally, emerging therapies based on NK cells, B cells, and macrophages, involving vaccines, cell modification, and so on. CAR‐T, chimeric antigen receptor‐T; CAR‐M, chimeric antigen receptor‐macrophage; DC‐CIK, dendritic cells (DC) combined with cytokine‐induced killer cells (CIK) IL‐2, interleukin‐2; IL‐12, interleukin‐12; TIM, tumor immune microenvironment. (Created with BioRender.com.)

As mentioned, exhausted T, NK, and B cells have been observed in cancer, characterized by impaired effector functions that drive tumor progression and poor outcomes. Currently, several mature strategies are available to reinvigorate exhausted NK and T cells, including ICIs and cytokine therapies. Chimeric antigen receptor (CAR)‐cell therapies can reverse individual immune exhaustion by introducing newly functional immune cells. Additionally, emerging therapies are being explored for their potential to reverse exhaustion. This section will review the current status and future prospects of these therapeutic approaches (Figure 5).

### 5.1 ICIs: The Expanding Therapeutic Armamentarium

Under normal physiological conditions, immune checkpoint molecules, including both inhibitory and stimulatory receptors, are constitutively expressed on immune cells to orchestrate immune homeostasis [196]. However, in tumors, overexpressed inhibitory receptors potently suppress the activation, proliferation, and effector functions of T and NK cells. ICIs counteract this by preventing the binding of these receptors to their corresponding ligands, thus reversing the exhausted state of T and NK lymphocytes. ICIs targeting PD‐1/programmed cell death protein ligand‐1 (PD‐L1), CTLA‐4, and LAG‐3 have advanced from research settings to become United States Food and Drug Administration (US FDA)‐approved clinical therapeutics. Further checkpoint targets, including TIM‐3, TIGIT, and NKG2A, are yielding encouraging outcomes in both preclinical investigations and clinical trials [197]. Emerging checkpoints like CD96, PVRIG, B7‐H3, VISTA, and BTLA are under development for therapeutic use [198].

*PD‐1/PD‐L1 Axis*. PD‐1 serves as the principal inhibitory checkpoint driving T cell exhaustion, first identified in studies of chronic LCMV infection [199]. Therapeutic blockade of PD‐1 axis can revitalize T cell effector function, as demonstrated in various cancers [200, 201, 202]. Given these therapeutic benefits, several PD‐1/PD‐L1 antagonists, including pembrolizumab, have received US FDA approval with promising results [203]. Notably, PD‐1 expression on NK cells is heterogeneous, varying across diseases contexts and even among healthy populations, where some individuals exhibit high PD‐1 expression [204]. In certain tumors, exhausted NK cells with high PD‐1 portends a poor prognosis. Blocking the PD‐1/PD‐L1 interaction proven effective in rescuing the functional dysfunction of these NK cells [94, 205].

*CTLA‐4*. Through high‐affinity binding to the shared ligands CD80/CD86, overexpressed CTLA‐4 outcompetes CD28 and thereby disrupts T cell proliferation and activation [206]. The first human anti‐CTLA‐4 inhibitor, ipilimumab, received approval in 2011 and initially demonstrated unexpected efficacy in melanoma [207]. Currently, the sole anti‐CTLA‐4 antibody licensed by the US FDA for clinical application is tremelimumab, exhibiting improved tolerance [208].

*LAG‐3*. Although broadly expressed on T, B, NK cells, and DCs, LAG‐3 has been most extensively studied in T cell biology [209]. Within tumors, it frequently co‐occurs with other inhibitory receptors on exhausted CD4^+^ and CD8^+^ T cells, a phenotype correlated with unfavorable clinical outcomes [210]. In various preclinical models, combined inhibition of LAG‐3 and PD‐1 yields superior tumor control compared with LAG‐3 blockade alone [211, 212].

*TIM‐3*. TIM‐3 is associated with exhausted CD8^+^ T and NK cells across multiple tumor types and acts to suppress these immune cells [213, 214, 215]. In liver tumors, blocking TIM‐3 reverses NK cell dysfunction, restoring their proliferation, killing capacity, and cytokine secretion [213]. Clinical trials of TIM‐3 inhibitors are underway, with accumulating evidence indicating that dual blockade of PD‐1/PD‐L1 and TIM‐3 provides better tumor control than single‐target blockade [197].

*TIGIT*. TIGIT shares the ligand CD155 with the costimulatory receptor DNAX accessory molecule‐1. Because it binds CD155 with higher affinity, TIGIT competitively delivers inhibitory signals that outweigh activation signals, thereby reducing T cell activation and proliferation. On NK cells, TIGIT serves as a particularly robust indicator of exhaustion. In tumor‐bearing mouse models, TIGIT blockade effectively preserves NK cell function from exhaustion and reinstates antitumor cytotoxicity [89]. Some studies suggest that antibodies targeting TIGIT can reverse the dysfunctional state of both T cells and NK cells [89]. Clinical trial results indicate that, with the exception of NSCLC, other cancer types have been investigated as potential targets for TIGIT blockade [216].

*NKG2A*. NKG2A is widely considered a critical immune checkpoint on NK cells. Its inhibition reinvigorates NK cell function across diverse cancer types, including both hematological and solid tumors [217, 218]. Notably, elevated NKG2A levels are also observed on exhausted CD8^+^ T cells, correlating with adverse prognosis in cancer patients [219]. NKG2A blockade has demonstrated enhanced functionality of CD8^+^ T cells [220].

Monotherapy targeting inhibitory receptors is associated with several limitations, including low response rates and significant adverse reactions. In contrast, combined blockade of multiple inhibitory receptors has demonstrated superior therapeutic outcomes. For example, dual blockade of CTLA‐4 and PD‐1 has shown enhanced efficacy in nail apparatus melanoma compared with PD‐1 monotherapy [221]. Furthermore, clinical trials have revealed promising results for various combinations of ICIs with surgery, chemotherapy, or targeted therapies. Beyond conventional treatment paradigms, the combined application of metabolic modulator and ICIs has also exhibited improved antitumor efficacy [222]. Notably, a meta‐analysis highlighted that combination therapies in HCC patients led to a significantly elevated incidence of immune‐related adverse events [223]. Therefore, further high‐quality studies are essential to optimize the clinical deployment of ICIs.

Although ICIs have achieved success in cancer immunotherapy and can provide sustained long‐term benefits, only a minority of patients exhibit significant responses. Therapeutic resistance is intrinsically connected to features of the TME [224], underscoring the need for deeper investigation into the cell‐level molecular mechanisms operative within it. Research has shown that this limited response is partly due to the proliferation of T_pex_ cells following ICI treatment, leading to an expansion of TEX^term^ cells [225]. Although TEX^term^ cells retain some degranulation capacity, their overall functionality remains restricted. In contrast, TEX^eff^ cells represent the most functionally competent subset within the exhausted T cell pool [37]. A promising therapeutic strategy involves redirecting T cell differentiation toward the TEX^eff^ subset rather than the TEX^term^ lineage. Nevertheless, the precise molecular circuitry that shapes divergent exhausted T cell fates remains unclear, and the functional role and definition of TEX^int^ cells require further elucidation. Addressing this gap necessitates a focused investigation into the heterogeneity of exhausted T cells to facilitate personalized tumor immunotherapy, particularly for patients who do not respond to conventional ICIs, as this may lead to significant therapeutic breakthroughs.

### 5.2 CAR‐T/NK Engineering: Precision and Limitations

Adoptive cell therapies (ACTs) enhance antitumor immunity by isolating a patient's immune cells, modifying and expanding them ex vivo, and reinfusing the resulting product [226]. The most prominent form, CAR‐T cell therapy, centers on engineering T lymphocytes to express a CAR. This synthetic receptor comprises an extracellular tumor–antigen‐binding domain and an intracellular signaling domain, enabling specific recognition and elimination of tumor [227]. In practice, patient‐derived T cells from peripheral blood are genetically modified via viral transduction to express the CAR, expanded to therapeutic doses in vitro, and then reinfused. Upon target engagement, these effector cells mediate tumor lysis predominantly by secreting perforin and granzyme B. Additionally, they secrete cytokines to recruit endogenous immune cells and can help establish persistent immunological memory against the malignancy.

In the last 10 years, CAR‐T cell therapy has demonstrated remarkable efficacy against hematological malignancies [228]. This success is underscored by regulatory approvals for multiple CAR‐T products, which achieve long‐term remission and even partial cures in certain cases [229]. In contrast,, translating this success to solid tumors presents major hurdles, including: (1) formidable inhibitory TME; (2) tumor antigen heterogeneity and antigen escape; and (3) severe potential side effects such as cytokine release syndrome (CRS), graft‐versus‐host disease (GVHD), and neurotoxicity [230]. To address these challenges, recent research efforts have explored various strategies, including enhancing CAR‐T cell potency, employing dual‐target therapies, and combining strategies to modulate the TME. For instance, Steffin et al. introduced IL‐15 into CAR‐T cells application, which yielded enhanced efficacy in patients compared with standard CAR‐T monotherapy [231]. These advances have opened up new prospects for the application of CAR‐T therapy to solid tumors [232].

NK cells exhibit potent cytotoxic effects against tumors, similar to T cells. Strikingly, NK cells have been demonstrated to resist tumor antigen escape and are linked to lower rates of CRS and GVHD [227]. These advantages establish NK cells as a compelling platform for CAR modification, offering a therapeutic alternative with improved safety and accessibility [233]. While CAR‐NK immunotherapy has proven highly effective against hematological cancers, its efficacy in solid tumors lacks robust clinical validation [233]. Significant barriers to success in solid tumors involve the limited lifespan of NK cells and immune suppression within the TME [227]. Key strategies include boosting the in vivo durability and proliferation of NK cells, with ongoing investigations exploring avenues such as the administration of immunostimulatory cytokines, genetic modification, and combinatorial therapies [234].

### 5.3 Cytokine Network Rebalancing

#### 5.3.1 Immune‐Stimulatory Paradigms

In the TME, multiple cell types upregulate immunosuppressive cytokines like TGF‐β and IL‐10. These cytokines recruit immunosuppressive cells, alter the phenotype and activity of effector lymphocytes, and reinforce an immunosuppressive niche. Hence, blocking inhibitory cytokines can help rejuvenate exhausted immune cells, thereby enhancing antitumor immune responses. Specifically, targeting TGF‐β inhibition augments CD8^+^ T cell‐mediated tumor clearance [235].

#### 5.3.2 Augmenting Stimulatory Cytokines

Supplementing with immune‐activating cytokines can help restore T and NK cell function. IL‐2 signaling supports T cell metabolic fitness and effector functions [236], and when combined with TIM‐3 blockade, it synergistically alleviates CD8^+^ T cell exhaustion [237]. IL‐15 is essential for NK cell maturation and activation [238]. Recombinant IL‐15 monotherapy effectively activated NK cells in cohorts of metastatic melanoma and renal cell carcinoma patients [239]. Additional cytokines, including IL‐21, IL‐7, IL‐17, and IL‐22, also hold immunotherapeutic potential, with several already under clinical evaluation [21, 240, 241, 242].

#### 5.3.3 Limitations and Advances

Cytokine therapies face challenges such as short half‐life and a narrow therapeutic window [243]. Strategies like PEGylation or antibody‐cytokine fusion proteins aim to prolong half‐life and improve tumor targeting, though further optimization is needed for precise and safe application. Some cytokines also exhibit dual roles, for example, IL‐2 can expand Treg cells, potentially counteracting effector T cell responses [244]. To address this, agents such as NKTR‐214 have been developed to preferentially signal through the IL‐2Rβγ receptor, stimulating CD8^+^ T and NK cells while avoiding the expansion of Tregs [245].

### 5.4 Emerging Therapeutic Frontiers

Current immunotherapies, including ICIs, CAR‐based therapies, and cytokine modulation, largely aim to reverse or bypass the exhausted state of T and NK cells. However, T cell exhaustion involves a complex interplay of metabolic, cellular, cytokine, and microbial factors, necessitating multifaceted therapeutic strategies. Several emerging approaches, used alone or alongside conventional treatments, have shown encouraging potential and are summarized in Table 2. Others, while promising, require further validation in clinical settings (Table 3).

**TABLE 2:** Emerging tumor immunotherapy used in published articles.

Immunotherapy	US FDA‐approved classic application	Promising combination therapy
TCR‐T	Afamitresgene autoleucel for advanced synovial sarcoma [246]	TCR‐T with IL‐21 receptor for hepatocellular carcinoma (HCC) model [247] TCR‐T with DC‐based vaccination for metastatic melanoma (MM) [248] TCR‐T with CAR‐T and panobinostat (epigenetic drugs) for pancreatic cancer (PC) model [249] TCR‐T with decitabine (chemotherapy) for acute myeloid leukemia (AML) model [250] TCR‐T with OVV‐01 (oncolytic virus) for liver cancer model [251] TCR‐T with atovaquone (ferroptosis‐targeted inducers) for HCC model [252] TCR‐T with tolinapant (immunomodulatory agent) for B cell acute lymphoblastic leukemia (B‐ALL) and melanoma [253]
TIL	Lifileucel for MM [254]	TIL with nivolumab (PD‐1 blockade) for metastatic osteosarcoma [255] TIL with vemurafenib (targeted drug) for MM [256] TIL with OV‐OX40L/IL12 (oncolytic virus) for solid tumor model [257] TIL with adjuvant chemotherapy for osteosarcoma [258] TIL with DC‐based vaccination for MM [259]
ICE	Blinatumomab for ALL [260]	CD33/CD16 with IL‐15 for AML model [261] HER2/FAP with nanomedicines for breast cancer (BC) [262] Mosunetuzumab (CD20xCD3) with lenalidomide (immunomodulatory agent) for follicular lymphoma [263] BiKE with CAR‐T and CAR‐NK for hematological malignancies and solid tumors model [264] Acasunlimab (PD‐L1×4‐1BB) with PD‐1 blockade for colon cancer (CC) model [265]
Tumor vaccine	Sipuleucel‐T for castration‐resistant prostate cancer [266]	IMA901 with cyclophosphamide (chemotherapy) for renal cell cancer [267] PRT/CpG/OVA nanovaccine with PD‐1 blockade for melanoma model [268] Tumor vaccine with lenvatinib (angiogenesis inhibitor) for oral squamous cell carcinoma model [269] CpG‐based tumor vaccine with radiation for lung carcinoma model [270] LRAST with anti‐Gr‐1 antibodies (MDSC depletion) for melanoma model [271] mRNA vaccine with nanoformulation for triple‐negative breast cancer model [272]
Oncolytic virus	Talimogene laherparepvec for advanced melanoma [273]	T‐Vec with ipilimumab (CTLA‐4 blockade) for advanced melanoma [274] oHSV with galunesertib (TGF‐β blockade) for glioblastoma [275] OVV‐Hyal1 with gemcitabine (chemotherapy) for PC model [276] G47Δwith RFA for HCC model [277] OVV with A56 CAR‐T for CC model [278] oHSV with trametinib (targeted drug) colorectal and lung carcinoma models [279]

Abbreviations: AML, acute myeloid leukemia; BC, breast cancer; BiKE, bispecific killer cell engager; B‐ALL, B cell acute lymphoblastic leukemia; CC, colon cancer; HER2, human epidermal growth factor receptor 2; ICE, immune cell engager; IL‑12, interleukin‑12; IL‑21, interleukin‑21; CAR‐NK, chimeric antigen receptor natural killer cells; CAR‑T, chimeric antigen receptor T cells; CpG, cytosine–phosphate–guanine oligodeoxynucleotide; CTLA‐4, cytotoxic T‐lymphocyte‐associated protein 4; DC, dendritic cells; FAP, fibroblast activation protein; HCC, hepatocellular carcinoma, LRAST, long peptide‑based adoptive T‑cell therapy; MM, metastatic melanoma; MDSC, myeloid‐derived suppressor cell; mRNA, messenger RNA; oHSV, oncolytic herpes simplex virus; OVA, ovalbumin; OVV, oncolytic vaccinia virus; PC, pancreatic cancer; PD‑1, programmed cell death protein 1; PD‐L1, programmed death‐ligand 1; PRT, personalized neoantigen; RFA, radiofrequency ablation; 4‐1BB, tumor necrosis factor receptor superfamily member 9; TIL, tumor‑infiltrating lymphocytes; TCR‑T, T‑cell receptor‑engineered T cells; TGF‑β, transforming growth factor‑beta.

**TABLE 3:** Emerging tumor immunotherapy in clinical trials.

Intervention	Study title	NCT number	Phases
CIK	Combined S‐1 with DC+CIK as maintenance therapy for advanced pancreatic ductal adenocarcinoma	NCT05955157	Phase 2/3
	Phase II study of chemotherapy and PD‐1 inhibitor combination with autologous CIK cell immunotherapy to treat lung cancer (CCICC‐002b)	NCT04836728	Phase 2
	The study of apatinib plus CIK as the third line therapy for advanced lung adenocarcinoma patients with wild‐type EGFR	NCT02493582	Phase 2
	Radiofrquency ablation combined with cytokine‐induced killer cells for the patients with cervical cancer	NCT02490748	Phase 2
DC vaccine	Platin‐based chemotherapeutics to enhance dendritic cell vaccine efficacy in melanoma patients	NCT02285413	Phase 2
	Neoantigen‐loaded DC vaccine, PD‐1 inhibitor, and radiotherapy for advanced NSCLC progressed after second‐line treatment	NCT06751849	Phase 2
	Basiliximab in treating patients with newly diagnosed glioblastoma multiforme undergoing targeted immunotherapy and temozolomide‐caused lymphopenia	NCT00626483	Phase 1
	Adoptive T cell therapy, DC vaccines, and hematopoietic stem cells combined with immune checkpoint blockade in patients with medulloblastoma (MATCHPOINT)	NCT06514898	Phase 1
Oncolytic virus	Clinical study of oncolytic virus in glioblastoma	NCT07145047	Phase 1/2
	Oncolytic virus in esophageal squamous cell carcinoma	NCT07061704	Phase 1/2
	TNFα and IL‐2 coding oncolytic adenovirus TILT‐123 with lymphocyte‐depleting chemotherapy and TILs in the treatment of melanoma (TUNINTIL‐2)	NCT06961786	Phase 1
	A study of recombinant oncolytic virus M1(VRT106) in patients with solid tumors	NCT06368921	Phase 1
	A clinical study on oncolytic virus injection (R130) for the treatment of advanced bone and soft tissue tumors	NCT06171282	Early Phase 1
ICE	Phase II study of anti‐PD‐1/VEGF bispecific antibody ivonescimab in patients with previously treated metastatic colorectal cancer	NCT06959550	Phase 2
	A study of teclistamab and mezigdomide in people with multiple myeloma	NCT07105059	Phase 1
	Study of BG‐T187 alone and in combination with other therapeutic agents in participants with advanced solid tumors	NCT06598800	Phase 1
	A study of JNJ‐80948543, a T‐cell redirecting CD79b×CD20×CD3 trispecific antibody, in participants with non‐Hodgkin lymphoma (NHL) and chronic lymphocytic leukemia (CLL)	NCT05424822	Phase 1
CAR‐γδT	Novel allogenic CD19‐targeting CAR‐γδT cell therapy in r/r NHL	NCT06838832	Phase 1/2
	Allogeneic CD7 CARγδT cells therapy recurrent/refractory leukemia	NCT07120607	Phase 1
	GPC3/mesothelin‐CAR‐γδT cells against cancers	NCT06196294	Phase 1
	Allogeneic γ9δ2 T cells treatment of recurrent hematologic tumors	NCT05755854	Phase 1
	Allogeneic B7H3 CAR‐γδT cell therapy for advanced solid tumors	NCT06825455	Early Phase 1
	γδT cell therapy for relapse prevention in high‐risk AML post‐transplant	NCT07123662	Early Phase 1
CAR‐NKT	A clinical research about CD70‐targeted CAR‐NKT cells therapy in subjects with advanced malignant solid tumors	NCT06728189	Phase 1
	Allogeneic NK T‐cells expressing CD19 specific CAR in B‐cell malignancies	NCT05487651	Phase 1
	GD2 specific CAR and interleukin‐15 expressing autologous NKT cells to treat children with neuroblastoma	NCT03294954	Phase 1
CAR‐DNT	RC1012 injection (allo‐DNT cells) for the prevention of relapse in AML patients after allo‐HSCT	NCT05858814	Phase 1/2
	Clinical study of the efficacy of CD19‐CAR‐DNT cells in the treatment of relapsed/refractory B‐cell NHL	NCT05453669	Phase 1
CAR‐M	Human HER2‐targeted macrophages therapy for HER2‐positive advanced gastric cancer with peritoneal metastases	NCT06224738	Early Phase 1
TIL	A multicenter, randomized, controlled, open label, phase II trial of autologous tumor infiltrating lymphocytes (GC101 TIL) in subjects with advanced melanoma (MIZAR‐003)	NCT06703398	Phase 2
	TIL therapy combined with pembrolizumab for advanced brain cancer including gliomas and meningiomas (BAH2472)	NCT06640582	Phase 1/2
	T‐cell therapy with CRISPR PD1‐edited tumor infiltrating lymphocytes for patients with metastatic melanoma (CRISPR‐TIL)	NCT06783270	Phase 1
	A study of gene‐edited GC203 TIL on the pancreatic ductal adenocarcinoma	NCT06828328	Early Phase 1
TCR‐T	E7 TCR‐T cell immunotherapy for human papillomavirus (HPV) associated cancers	NCT05686226	Phase 2
	A clinical study of multitarget Hi‐TCR‐T cells in the treatment of advanced hepatocellular carcinoma	NCT06902389	Phase 1/2
	Autologous HBV‐TCR T cell therapy (LioCyx‐M) for the treatment of hepatocellular carcinoma	NCT06961617	Phase 1
	An open‐label, phase I clinical trial of super1 TCR‐T in NY‐ESO‐1‐positive patients with advanced solid tumors	NCT06942143	Phase 1

*Data source*: ClinicalTrials.gov (U.S. National Library of Medicine). Data accessed on September 28, 2025.

*Abbreviations*: CIK, cytokine‐induced killer cells; CAR‐γδT, chimeric antigen receptor‐engineered gamma delta T cells; CAR‐NKT, chimeric antigen receptor‐engineered natural killer T cells; CAR‐DNT, chimeric antigen receptor‐engineered double‐negative T (CD4^−^CD8^−^) cells; CAR‐M, chimeric antigen receptor‐engineered macrophages; TCR‑T, T‑cell receptor‑engineered T cells; DC, dendritic cells; PD‐1, programmed cell death protein 1; EGFR, epidermal growth factor receptor; EGFR, epidermal growth factor receptor; TNF‐α, tumor necrosis factor‐alpha; IL‐2, interleukin‐2; TIL, tumor‐infiltrating lymphocytes; VEGF, vascular endothelial growth factor; B7H3, B7 homolog 3 protein; AML, acute myeloid leukemia; allo‐HSCT, allogeneic hematopoietic stem cell transplantation; HER2, human epidermal growth factor receptor 2; CRISPR, clustered regularly interspaced short palindromic repeats; HBV, hepatitis B virus.

#### 5.4.1 Engineering the Immune Response: The ACTs Platform Revolution

**FIGURE 6:**
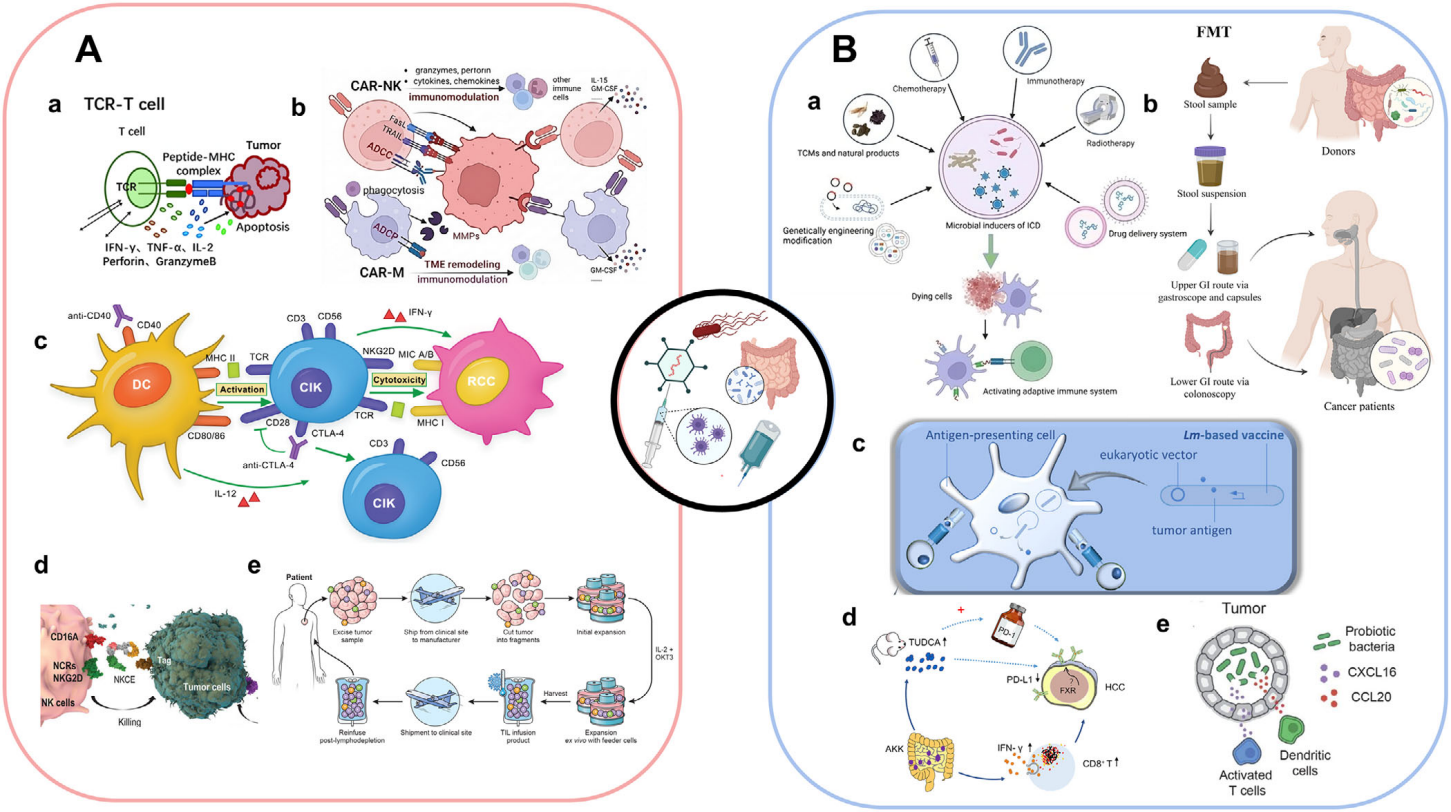
Other tumor immunotherapy methods. (A) Immune cells‐based tumor immunotherapy. Copyright 2023 by Li [331], 2023 by Liu [332], 2022 by Zhang [333], 2021 by Demaria [334], 2025 by Turcotte [335]. (B) Microorganism‐based tumor immunotherapy. Copyright 2024 by Huang [336], 2024 by Yang [337], 2021 by Oladejo [338], 2024 by Lan [339], and 2023 by Savage [340].

Beyond CAR‐T and CAR‐NK therapies, multiple ACT modalities are demonstrating clinical potential across various cancers (Figure 6A). Among these, CAR‐T, tumor infiltrating lymphocyte (TIL), and TCR‐T therapies have received US FDA approval. TILs recognize a broad spectrum of tumor antigens and exhibit robust tumor‐homing capability, helping to mitigate problems of tumor heterogeneity and poor T cell infiltration. However, TIL therapy encounters production hurdles, notably in identifying and isolating reactive TIL subsets [280]. TCR‐T therapy involves genetically programming T cells to express TCRs targeting MHC‐presented tumor antigens [281]. Despite dilemma such as TCR mismatch and potential toxicity, TCR‐T therapies show broader applicability in solid tumors compared with CAR‐T approaches [282].

While the majority of CAR‐T products are manufactured from peripheral blood mononuclear cells (PBMCs), studies indicate that specific T cell subpopulations may offer enhanced antitumor efficacy compared with mixed populations [283]. Consequently, alternative cell sources such as γδT cells, invariant NK T cells, and double‐negative T cells are under exploration [284]. The constraints imposed by the TME and ECM on CAR‐T and CAR‐NK efficacy have prompted the exploration of CAR‐macrophages as a promising alternative for treating solid tumors. These cells not only infiltrate the TME effectively but also modulate immunosuppressive conditions and remodel the ECM [285, 286]. Cytokine‐induced killer (CIK) cells are a heterogeneous population derived from PBMCs through in vitro stimulation with anti‐CD3 antibodies, IL‐2, and IFN‐γ [226]. They exhibit potent MHC‐independent antitumor activity, combining the functional features inherent to NK cells, NKT cells, and T cells [287].

#### 5.4.2 Tumor Vaccines: A Dual Front Against Cancer From Prophylaxis to Therapy

Tumor vaccines are classified as either therapeutic or preventive [288]. Therapeutic versions deliver tumor‐associated antigens or tumor‐specific antigens (TSAs) to APCs, which subsequently activate T cells to mount antitumor immune responses [289]. Approved therapeutic vaccines include sipuleucel‐T for castration‐resistant prostate cancer and Bacillus Calmette–Guerin vaccine for high‐risk nonmuscle‐invasive bladder cancer [290]. DC‐based vaccines represent a distinct approach, in which DCs loaded with TSAs are reinfused to stimulate T cell immunity [291]. Preventive vaccines target oncogenic viruses to block infection and subsequent carcinogenesis [292]. Examples include the HPV vaccine Cervarix, approved in 2009, which prevents HPV‐related cancers [293], and the HBV vaccine, which reduces liver cancer risk [294]. Vaccines against other oncogenic viruses such as EBV and HCV are under development [295].

#### 5.4.3 Oncolytic Viruses

Oncolytic viruses (OVs) constitute a novel therapeutic modality for cancer, capable of inducing direct oncolysis while stimulating host antitumor immunity for long‐term control [296]. OVs have also shown potential in reversing T cell exhaustion. Conditionally replicating adenoviruses can significantly decrease PD‐1 levels on tumor‐infiltrating CD8^+^ and CD4^+^ T cells [297].

#### 5.4.4 Precision Targeting: Novel Therapeutic Horizons

Metabolic alterations, primarily including mitochondrial dysfunction, amino acid restriction, accumulation of reactive oxygen species, and dysregulated lipid metabolism, have been observed in exhausted CD8^+^ T cells [55]. Various transcription factors, including TCF‐1, TOX, Eomes, T‐bet, IRF4, BATF, and NFAT, are involved in the establishment and maintenance of T cell exhaustion [32]. Additionally, exhausted T lymphocytes undergo epigenetic modifications. For example, promoter demethylation of PD‐1 leads to upregulation of the inhibitory receptor PD‐1 [298]. Targeting metabolic processes, specific transcription factors, and epigenetic modifications can reverse T cell exhaustion. Antioxidants targeting mitochondrial counteracts T cell exhaustion and have been validated in antitumor immunotherapy [299]. Shan et al. induced TCF‐1 expression in exhausted T cells, driving a shift toward a T_pex_ state, significantly enhancing their response potential [300]. L‐2‐HG, through epigenetic modification of Tex cells, reduces TOX expression and enhances the antitumor activity of T cells [301].

#### 5.4.5 Next‐Generation Immune Cell Engagers: Integrating Checkpoint Blockade

Immune cell engagers (ICE) serve as bridges connecting immune cells to cancer cells, typically constructed as bispecific antibodies. One arm is engineered to recruit T cells via CD3 and NK cells via CD16 or NKG2D, while the other targets TSAs to trigger effective tumor‐targeted cytotoxicity [302]. By incorporating checkpoint‐targeting domains into ICE, specific blockade of inhibitory ligands on tumor cells can be achieved without affecting other cell types. For example, a PD‐1×αCD3×αCD33 trispecific antibody selectively lyses PD‐L1^+^ AML cells [303], and an EGFR×CD16a×PD‐L1 trispecific antibody enhances NK cell‐mediated ADCC against EGFR^+^PD‐L1^+^ tumors [304]. Given their precise targeting capability, ICEs may help prevent T cell and NK cell exhaustion in vivo, representing a compelling direction for future research.

#### 5.4.6 Harnessing Nonimmune Players

Beyond immune cells, the microbiota and its metabolites influence the immune microenvironment [305], inspiring microbial‐based immunotherapies such as fecal microbiota transplantation, attenuated bacterial vaccines, and engineered microorganisms (Figure 6B).

Additional strategies, like intelligent nanoparticles for targeted delivery and induction of ferroptosis in tumor cells, can also reshape the immune landscape and synergize with ICIs [306, 307]. Although not directly targeting immune cells, these approaches contribute to the overall efficacy of cancer immunotherapy. Comprehensively mapping the interactions among immune cells and microenvironmental cues will support the rational integration of emerging modalities to overcome immune exhaustion and improve treatment outcomes.

## 6 Therapeutic Strategies Targeting Immune Cell Exhaustion in Chronic Infection

### 6.1 The Heterogeneity of Exhaustion: Toward Precision ICI Therapy for Chronic Infection

ICIs have become a cornerstone of modern oncology. Given that pathogens exploit the upregulated ICs to facilitate immune escape in chronic infections, ICIs may also hold promise for controlling infections and improving patient outcomes. For example, PD‐1/PD‐L1 blockade augments antifungal defenses and improves outcomes in mouse models of invasive pulmonary aspergillosis [308]. Furthermore, during chronic viral infections, checkpoint blockade in vitro reduces viral load and restores T‐cell function [29]. Although preclinical studies underscore the potential of ICIs in treating infectious diseases, clinical translation remains challenging due to insufficient evidence regarding their safety and efficacy in these settings.

Heterogeneity in inhibitory receptor expression among different pathogens and even within the same infection during disease progression underscores the complexities in treating infections with ICIs. For instance, in HTLV‐1 chronic infection, PD‐1 levels are linked to CD8^+^ T cell functionality and viral load [309]. In contrast, there appears to be no notable correlation between the levels of TIM‐3, LAG‐3, and CTLA‐4 and these factors [136]. The evolving landscape of inhibitory receptor expression in chronic infections provides a compelling rationale for the development of combination therapies. Such approaches could potentially target multiple inhibitory pathways simultaneously, addressing the heterogeneity and complexity of the immune microenvironment. For example, in chronic LCMV infection, blocking LAG‐3, TIM‐3, or TIGIT alone showed limited improvements, whereas coblockade with PD‐1 yielded superior outcomes [310]. Future studies should identify biomarkers that predict effective ICI combinations for specific patient populations to enable precision therapy.

### 6.2 CAR‐T/NK: A Potential Antiviral Platform

Although as a critical strategy in tumor immunotherapy, the applications of CAR‐cells have expanded to viral infections. Modified design of CARs allows CAR‐T or CAR‐NK cells to target virus‐infected cells, thereby enhancing their cytotoxic activity. CAR‐T therapies have demonstrated robust antiviral activity against infections caused by severe acute respiratory syndrome coronavirus 2, HIV, HBV, HCV, human cytomegalovirus, and EBV [311, 312]. Notably, they demonstrate superior therapeutic efficacy against HIV, primarily due to their ability to recognize antigens independent of MHC‐I, which is downregulated by HIV [313].

CAR‐NK cells also exhibit potential for controlling viral infections. Lim et al. developed CAR‐NK cells targeting multiple epitopes of HIV gp160, overcoming viral diversity and supporting viral clearance [314]. While still emerging, this approach may build on targets already validated in CAR‐T studies. Other adoptive therapies, such as induced pluripotent stem cell‐derived HIV‐specific cytotoxic T cells, have also shown sustained antiviral efficacy [315].

### 6.3 Targeting Exhaustion: Synergistic Approaches in Chronic Infection

Cytokine network dysregulation in chronic infection provides another therapeutic avenue. Blocking inhibitory cytokines or administering stimulatory cytokines can restore immune cell function. In HIV infection, IL‐10 blockade increases IFN‐γ secretion from CD4^+^ T cells and diminishes viral load [316]. IL‐2 counteracts PD‐1‐mediated inhibition in vitro, and recombinant IL‐2 enhances effector function of HBV‐specific CD8^+^ T cells in vivo [317, 318]. In HBV infection, IL‐12 improves CD8^+^ T cell function and synergizes with PD‐1 blockade [319].

Exhausted T cells in chronic infection also display metabolic alterations that can be targeted therapeutically. In HBV and HCV infection, enolase acts as a critical metabolic regulator in exhausted CD8^+^ T cells; overcoming this constraint revitalizes antiviral function [320].

### 6.4 Pathogen Reduction: Alleviating the Burden of Exhaustion

Since exhaustion is largely driven by persistent antigen, reducing pathogen load may reverse the dysfunctional state. Isoniazid preventive therapy for latent Mtb infection downregulates PD‐1 on pathogen‐specific T cells, indicating reduced exhaustion following decreased bacterial burden [321]. Similarly, partial functional recovery of HCV‐specific CD8^+^ T cells has been reported following IFN‐free treatment of chronic HCV infection [322]. A notable exception is monoclonal antibody therapy: although capable of neutralizing viral antigens, it fails to reliably induce a sustained virologic response on its own [323]. Instead, its additional immunomodulatory effects, mediated through Fc/Fc receptor interactions with immune cells, led to reduced PD‐1 expression on both CD4^+^ and CD8^+^ T cells in HIV infection, thereby enhancing T cell function [324]. These findings collectively indicate that reducing pathogen load holds promise for alleviating T cell exhaustion, although its efficacy depends on the pathogen type and treatment modality, underscoring the need for further research in this area.

### 6.5 Epigenetic Reprogramming: Toward a Definitive Eradication

Following virus eradication mediated by direct‐acting antivirals (DAAs) in hepatitis, the withdrawal of persistent antigen stimulation leads to the disappearance of Tex^term^ cells. However, exhausted core signaling can still be detected in T_pex_ cells [325]. Subsequent studies have demonstrated that chronic antigen exposure imprints an epigenetic scar on exhausted CD8^+^ T cells, which persists even after infection resolution [326]. In addition to DAAs, ICIs also fail to fundamentally reverse these exhaustion‐related epigenetic alterations [68]. Therefore, achieving complete reversal of T cell exhaustion may require therapeutic strategies directly targeting epigenetic programming.

## 7 Chronic Infection–Immune Exhaustion–Tumor Axis: Pathways and Treatment

The interplay between chronic viral infection and tumorigenesis has been attributed to viral oncogenicity and immune evasion. However, emerging evidence highlights that chronic infection, distinct from acute or noninfectious states, induces profound immunosuppression that drives tumor initiation, progression, therapy resistance, and poor prognosis. This phenomenon is not limited to viruses; it has also been observed in other pathogens, such as fungi, bacteria, and helminths. Central to this process is immune cell exhaustion, particularly of T cells, which is a dysfunctional state observed across chronic infections and malignancies. Notably, exhausted T cells in these contexts exhibit distinct molecular signatures, inspiring us to further explore the different roles of exhaustion in disease networks [24]. While few studies have systematically explored this chronic infection‐exhaustion‐tumor triad, we propose a dual‐phase model: pretumorigenic immune exhaustion fosters oncogenesis, while post‐tumorigenic immunosuppressive microenvironments accelerate disease progression and undermine immunotherapy efficacy. Validating this paradigm requires robust clinical trials to elucidate causal relationships and therapeutic implications.

Significant new insights have emerged in the field of tumor immunotherapy, including prophylactically reducing tumorigenesis and mitigating its pathological progression. Primary prevention of infection or preventing pathogen immune escape into chronicity represents a critical initial step in targeting this chronic infection–exhaustion–tumor axis. Thus, prophylactic vaccines and antimicrobial therapies serve as foundational interventions. Notably, prophylactic antiviral vaccination has demonstrated potential in preventing virus‐associated carcinogenesis. There is an urgent need to expand the development of vaccines, particularly against pathogens with established oncogenic potential. Unfortunately, complete eradication of chronic infections is often unfeasible. Therefore, intervention in the ensuing immunosuppressive environment presents an alternative strategy for enhancing outcomes in tumor patients. Specifically, as described above, controlling or reversing immune exhaustion during chronic infection may prevent the progression from chronic infection to malignancy. Furthermore, in cancer patients with comorbid chronic infections, active anti‐infection treatment coupled with reversal of T cell exhaustion should be implemented to mitigate cancer progression. Finally, ICIs play an indispensable role: they not only directly target tumors but also proactively manage premalignant chronic infection states, making it an outstanding weapon in tumor immunotherapy.

## 8 Discussion

Recent advances have revealed that T cell exhaustion represents an impaired yet salvageable functional state, highlighting the importance of understanding its molecular drivers. The diverse exhaustion states of CD8^+^ T cells determine their efficacy in immunotherapy and infection control, necessitating further breakthroughs at the transcriptional and epigenetic levels. Current knowledge about CD4^+^ T cell exhaustion often relies on concepts derived from CD8^+^ T cells, overlooking CD4^+^ plasticity and subset diversity [327]. Future work should characterize exhaustion across CD4^+^ subsets in infection and cancer, and assess their therapeutic responses. Current mainstream therapeutic strategies to combat exhaustion, including ICIs, CAR‐T therapy, and cytokine modulation, have demonstrated substantial clinical benefits. Emerging evidence highlights the metabolic–epigenetic axis, where metabolic stress and effector gene silencing interact, as a promising new target for intervention, reflecting more precise strategies for reinvigorating exhausted T cells. Additionally, given the heterogeneity of T cell exhaustion across subsets and disease contexts, future efforts should prioritize multimodal combination therapies that target diverse signaling pathways involved in exhaustion, thereby refining therapeutic strategies [328].

Given their pivotal contributions to both anti‐infection and antitumor immunity, NK cell exhaustion has gradually garnered increasing attention. However, the mechanistic basis and reversal strategies for NK cell exhaustion remain far less understood relative to T cells. This knowledge gap stems in part from the phenotypic and functional overlap between exhaustion and other types of NK cell dysfunction, such as anergy [329]. Moreover, NK cells exhibit divergent exhaustion features in chronic infection versus tumor, suggesting distinct mechanistic underpinnings that warrant further in‐depth investigation [330].

While contemporary immunotherapies targeting exhausted immune cells offer promising therapeutic avenues, their clinical application faces multifaceted challenges, including phenotypic heterogeneity of exhausted populations, intricate cytokine crosstalk, substantial implementation costs, and unresolved safety profiles. To optimize therapeutic decision‐making, a comprehensive investigation of the infection–exhaustion–oncogenesis axis should be integrated with patient‐specific immune contextualization through advanced immunological profiling. Notably, comparative pathobiological analyses reveal distinct mechanistic signatures between infection‐induced and tumor‐associated immune exhaustion, a critical differentiation demanding rigorous characterization. This biological dichotomy necessitates dual investigative imperatives: (1) systematic elucidation of disease‐specific exhaustion pathways through multiomics approaches, and (2) parallel development of context‐adapted immunotherapeutic strategies that balance clinical efficacy with safety parameters. Such targeted research initiatives could enable earlier intervention windows and more precise modulation of tumor‐related immune dysfunction.

## Author Contributions

Writing – original draft: YLS and YZM. Funding acquisition: WDZ, YLS, YH, and WW. Writing – review, editing, and visualization: SC. Visualization: YMC and CYZ. Formal analysis and investigation: YMC and SYD. Data curation and investigation: JL. Resources and investigation: YH and WW. Conceptualization and supervision: TTZ and WDZ. Project administration: TTZ and WDZ. All authors have read and approved the final manuscript.

## Funding

This study was supported in part by National Natural Science Foundation of China (No. 82302632, Yali Song), Natural Science Foundation of Sichuan Province (No. 2025ZNSFSC0550, Yali Song), Shenzhen Science and Technology Innovation Commission (No. JCYJ20220531102401003, Weidong Zheng), the grants from the Clinical Research Center for Gastrointestinal Cancer in Hunan Province (No. 2021SK4016, Yi He and Wei Wang), and Climbing Fund of the National Cancer Center (No. NCC201909B05, Yi He).

## Conflicts of Interest

The authors declare no conflicts of interest.

## Ethics Statement

The authors have nothing to report.

## Data Availability

The authors have nothing to report.
